# Supplementary figures and images for: Using symptom-based case predictions to identify host genetic factors that contribute to COVID-19 susceptibility
Source: PLoS One. 2021 Aug 11;16(8):e0255402. doi: 10.1371/journal.pone.0255402 (PMC8357137; doi:10.1371/journal.pone.0255402)

A: rs11844522

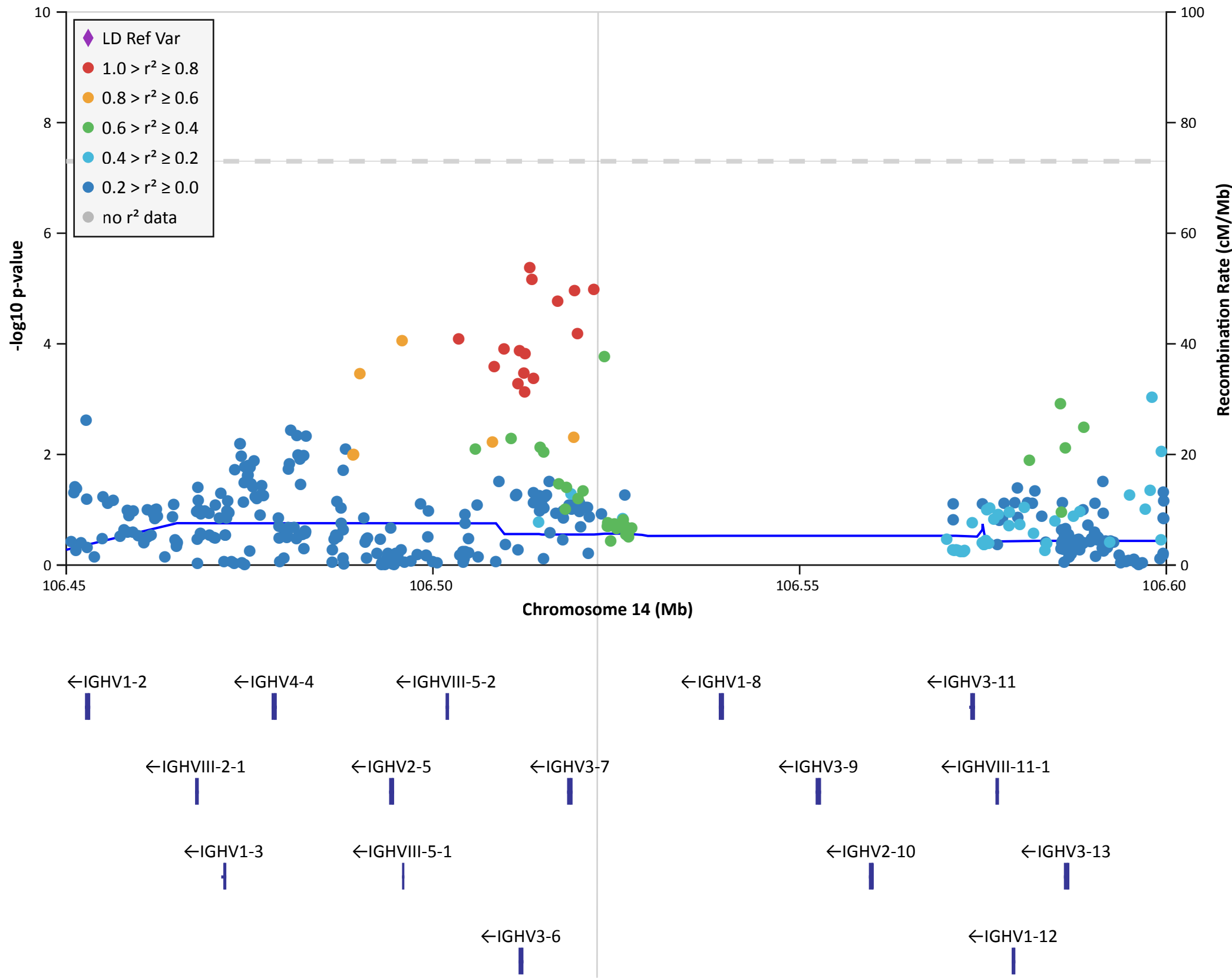

B: rs5798227

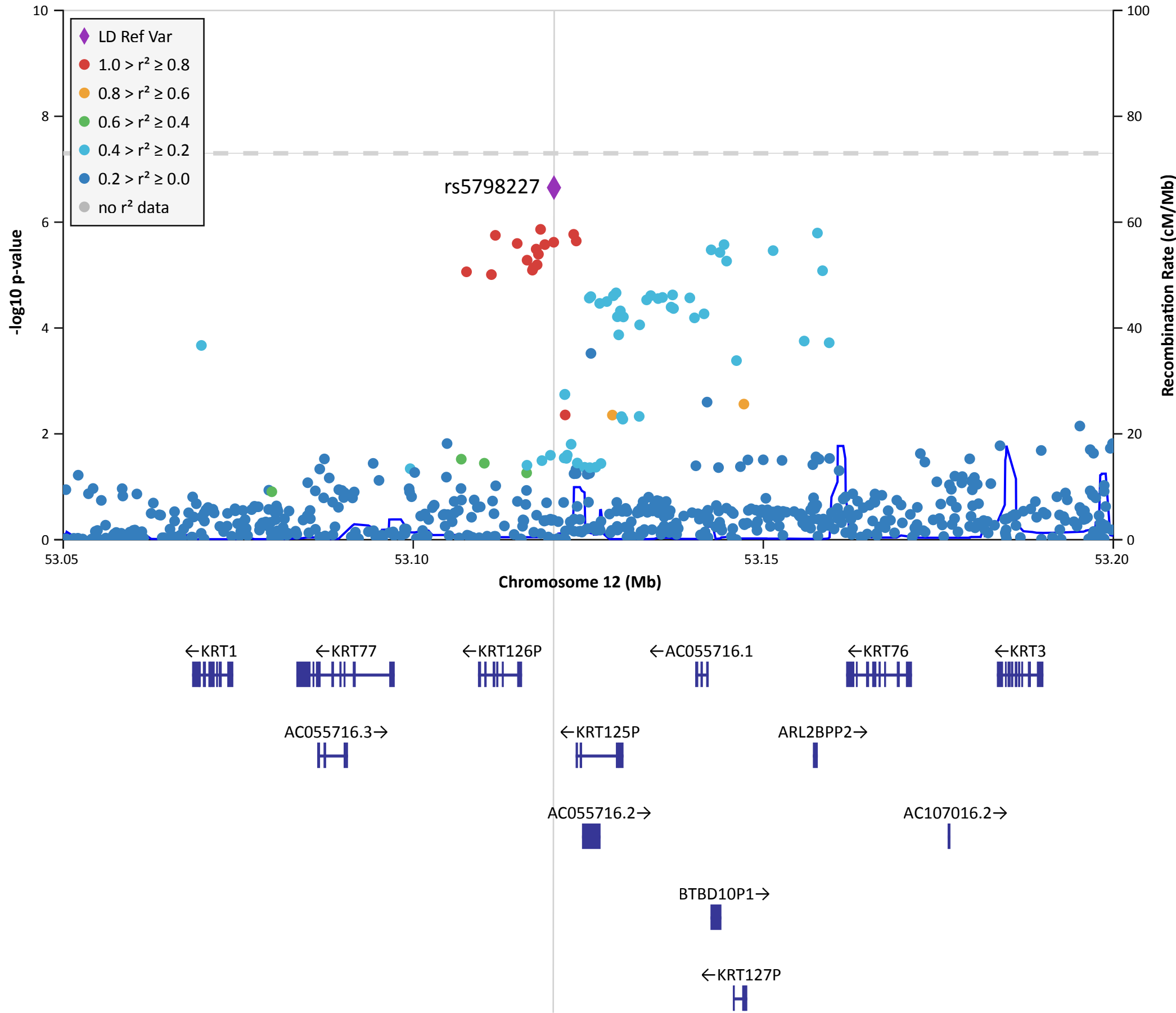

Supplement: S1 Fig — In each of the two panels, the top SNP is indicated by a purple diamond. Other SNPs are colored according to their linkage disequilibrium with the top SNP (calculated with the European population from the 1000 Genomes Project (phase 3) as a reference panel). The genes located within the visualized regions are drawn at their respective locations, with an arrow indicating the transcribed strand. Positions correspond to genome assembly GRCh37. (PDF) [file pone.0255402.s001.pdf]

# Enrichment of common diseases among predicted COVID-19 cases and positively tested COVID-19 cases

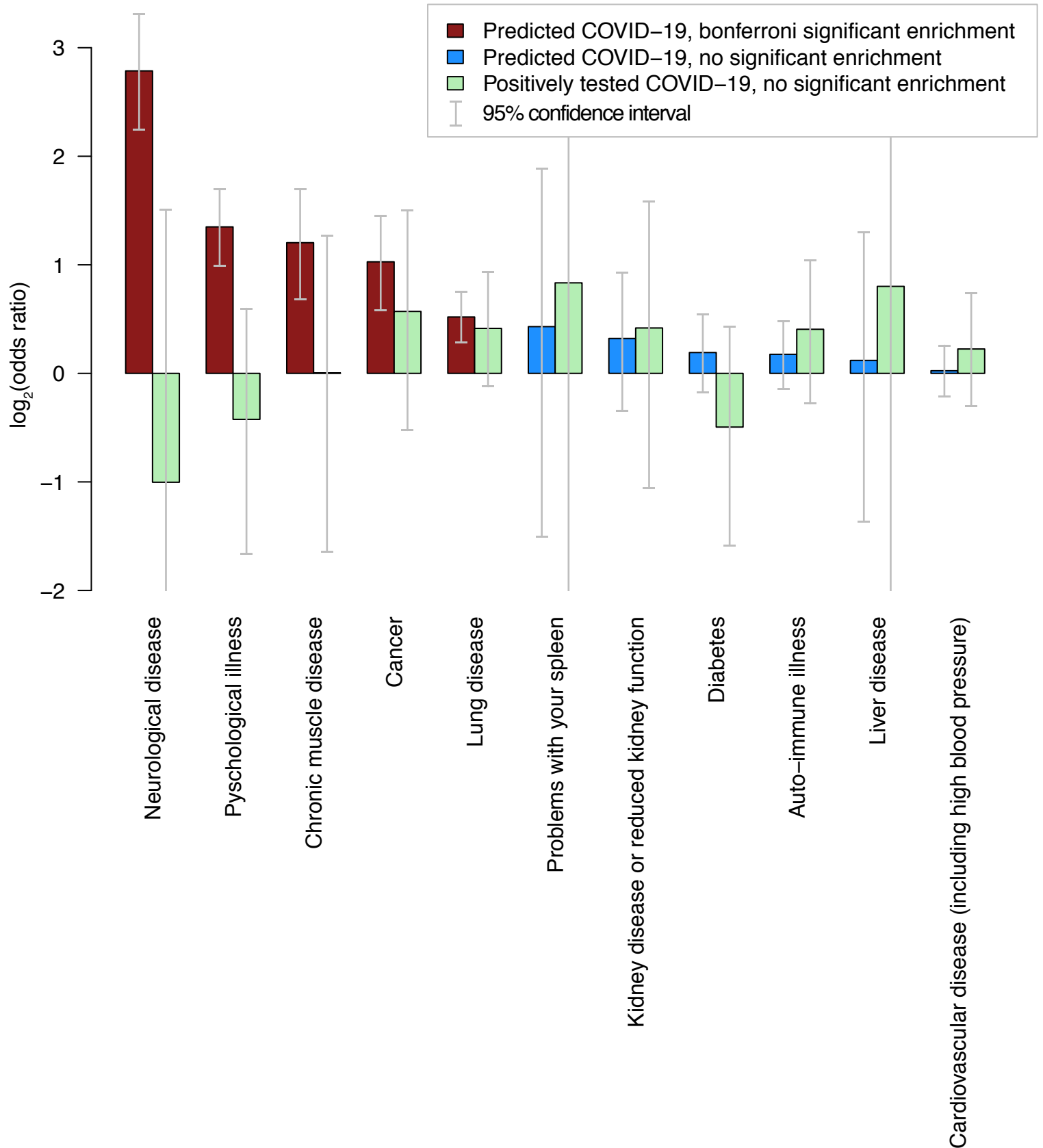

Supplement: S4 Fig — Fisher’s exact test shows a Bonferroni significant positive correlation between “Neurological disease”, “Psychological disease”, “Chronic muscle disease”, “Cancer” and “Lung disease” patients and COVID-19 predicted cases. This association is not present for positive COVID-19 cases. Applying generalised linear models with “age”, “sex” and “bmi” as covariates, instead of Fishers exact tests, does not change which diseases are Bonferroni significant and which are not. (PDF) [file pone.0255402.s004.pdf]
