# Supplementary material for: Using symptom-based case predictions to identify host genetic factors that contribute to COVID-19 susceptibility
Source: PLoS One. 2021 Aug 11;16(8):e0255402. doi: 10.1371/journal.pone.0255402 (PMC8357137; doi:10.1371/journal.pone.0255402)

A: hepatitis B virus infection

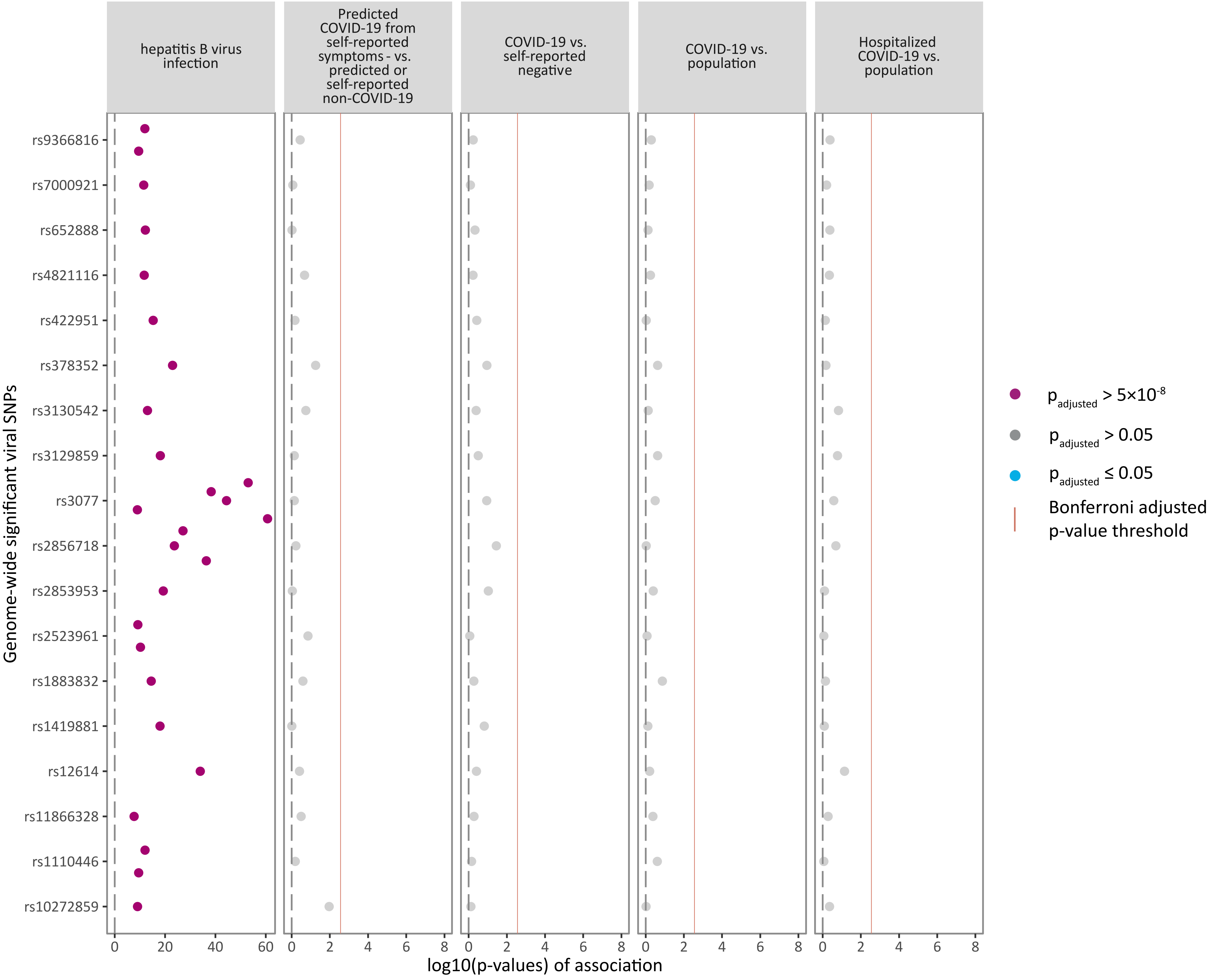

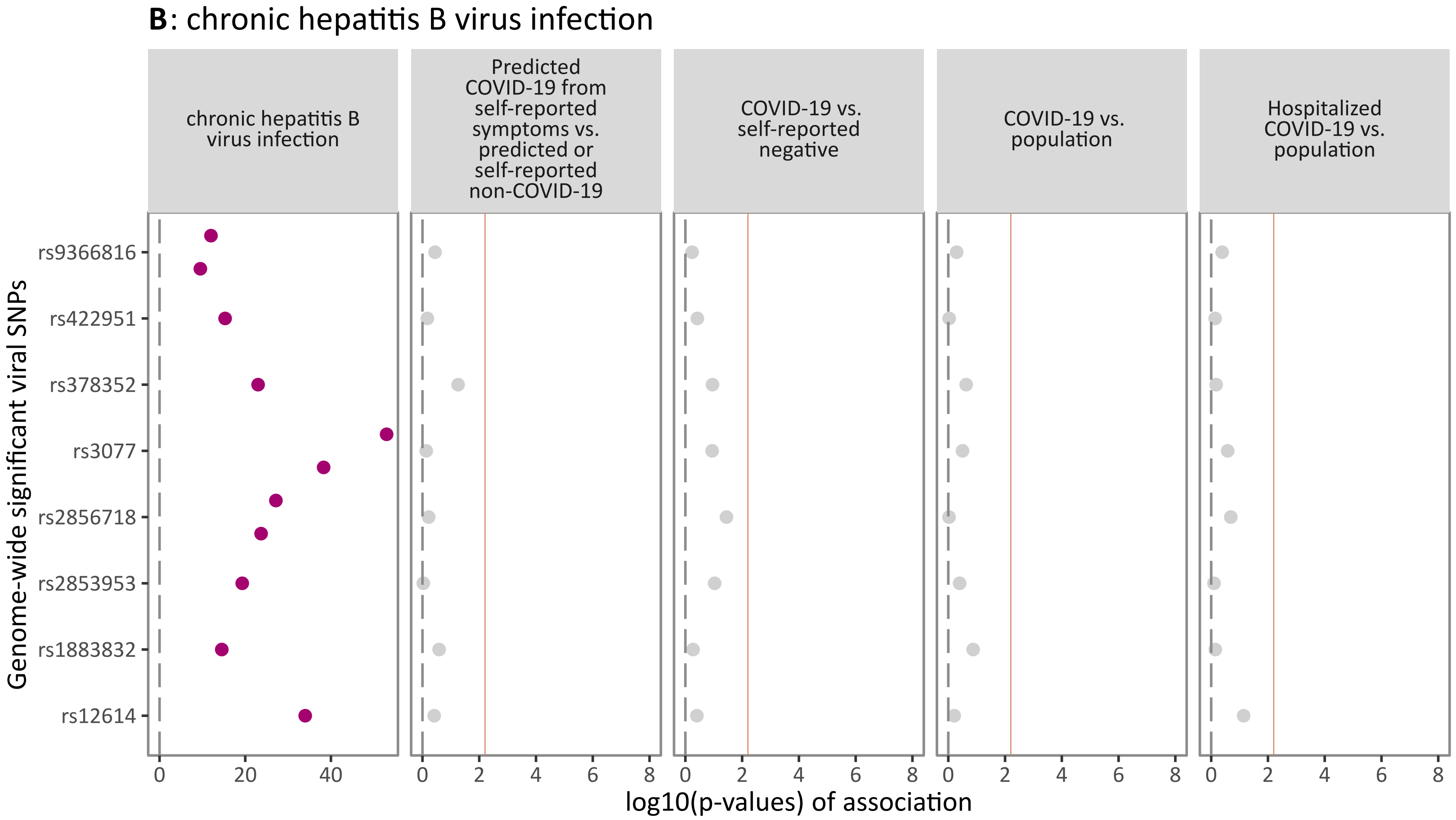
B: chronic hepatitis B virus infection
chronic hepatitis B virus infection
Predicted COVID-19 from self-reported symptoms vs. predicted or self-reported non-COVID-19
COVID-19 vs. self-reported negative
COVID-19 vs. population
Hospitalized COVID-19 vs. population
Genome-wide significant viral SNPs
rs9366816
rs422951
rs378352
rs3077
rs2856718
rs2853953
rs1883832
rs12614
0
20
40
0
2
4
6
8
log10(p-values) of association

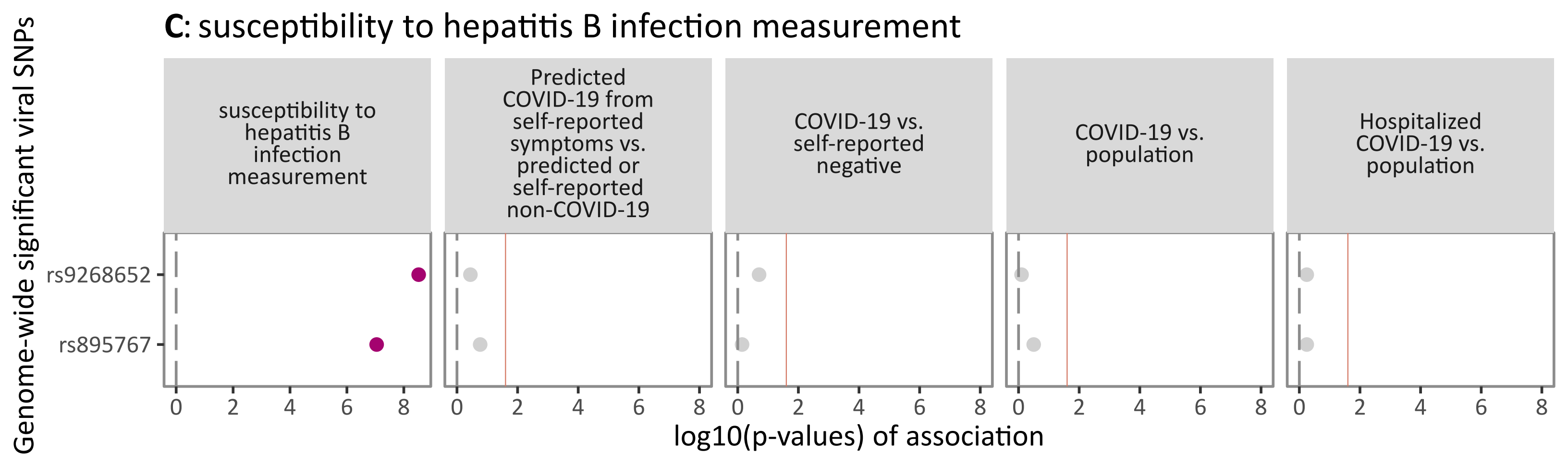

C: susceptibility to hepatitis B infection measurement
susceptibility to hepatitis B infection measurement
Predicted COVID-19 from self-reported symptoms vs. predicted or self-reported non-COVID-19
COVID-19 vs. self-reported negative
COVID-19 vs. population
Hospitalized COVID-19 vs. population
Genome-wide significant viral SNPs
rs9268652
rs895767
0
2
4
6
8
log10(p-values) of association

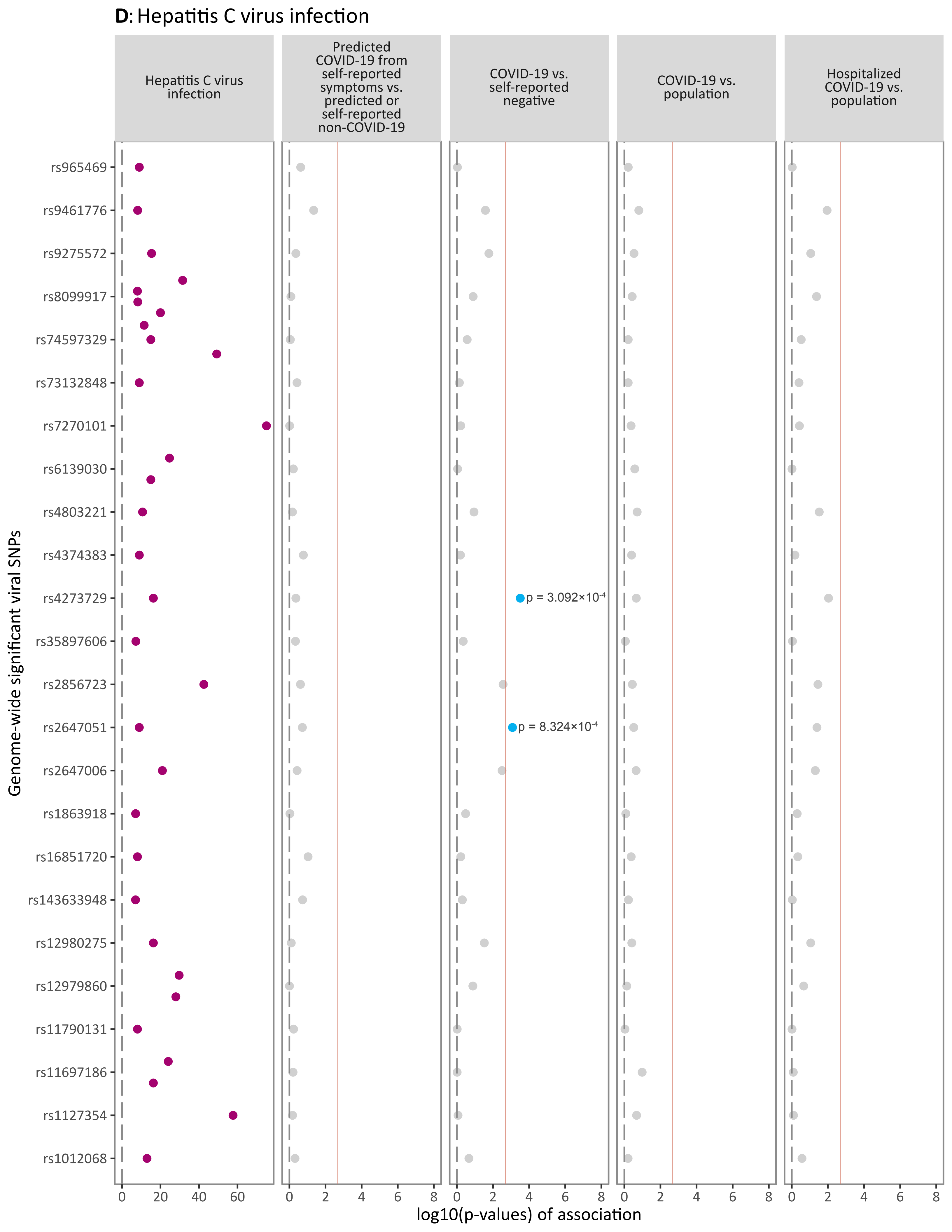
D: Hepatitis C virus infection
Hepatitis C virus infection
Predicted COVID-19 from self-reported symptoms vs. predicted or self-reported non-COVID-19
COVID-19 vs. self-reported negative
COVID-19 vs. population
Hospitalized COVID-19 vs. population
rs965469
rs9461776
rs9275572
rs8099917
rs74597329
rs73132848
rs7270101
rs6139030
rs4803221
rs4374383
rs4273729
rs35897606
rs2856723
rs2647051
rs2647006
rs1863918
rs16851720
rs143633948
rs12980275
rs12979860
rs11790131
rs11697186
rs1127354
rs1012068
p = 3.092×10-4
p = 8.324×10-4
Genome-wide significant viral SNPs
log10(p-values) of association
0 20 40 60
0 2 4 6 8

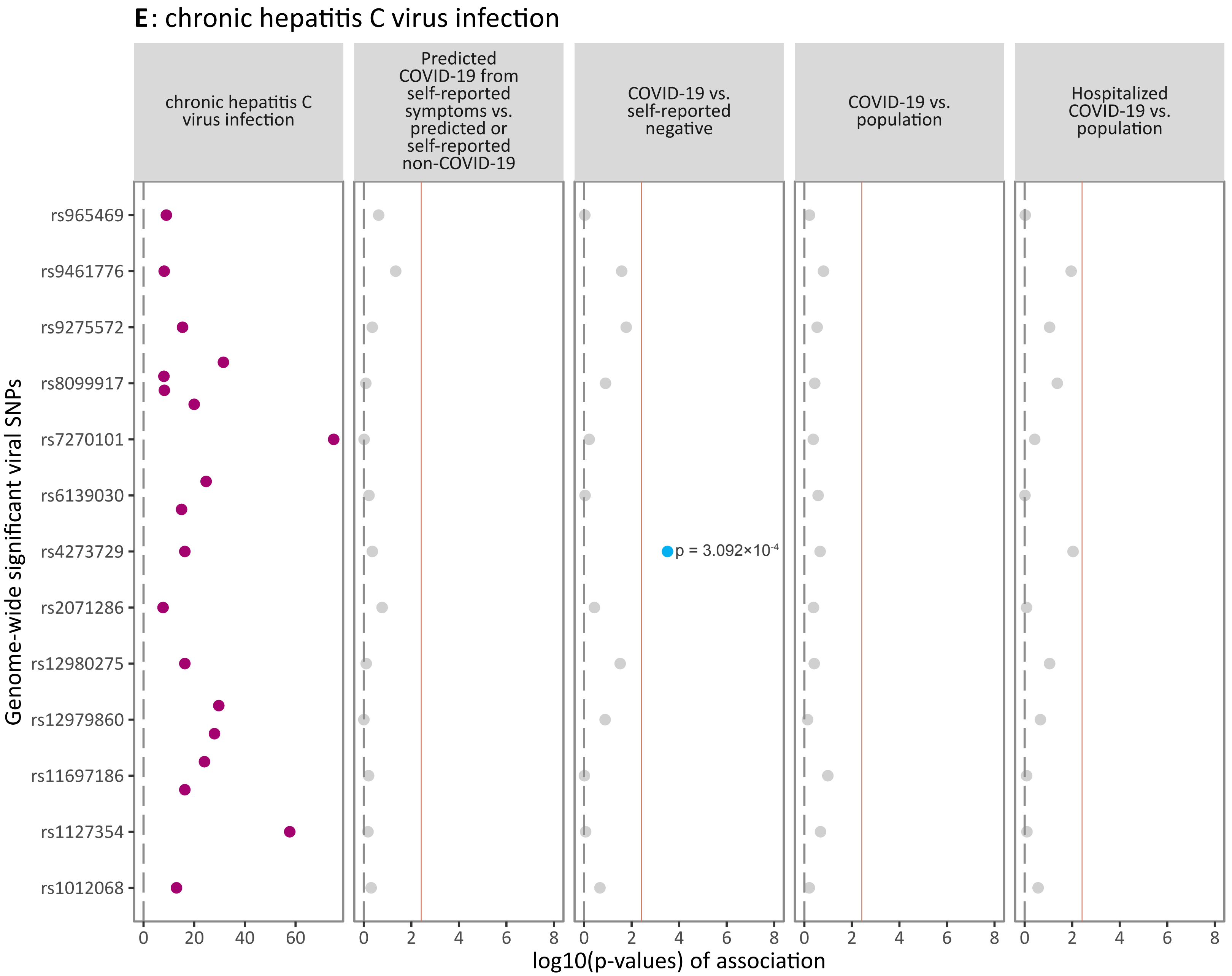

E: chronic hepatitis C virus infection
chronic hepatitis C virus infection
Predicted COVID-19 from self-reported symptoms vs. predicted or self-reported non-COVID-19
COVID-19 vs. self-reported negative
COVID-19 vs. population
Hospitalized COVID-19 vs. population
rs965469
rs9461776
rs9275572
rs8099917
rs7270101
rs6139030
rs4273729
rs2071286
rs12980275
rs12979860
rs11697186
rs1127354
rs1012068
p = 3.092×10-4
0
20
40
60
2
4
6
8
Genome-wide significant viral SNPs
log10(p-values) of association

F: Epstein-Barr infection

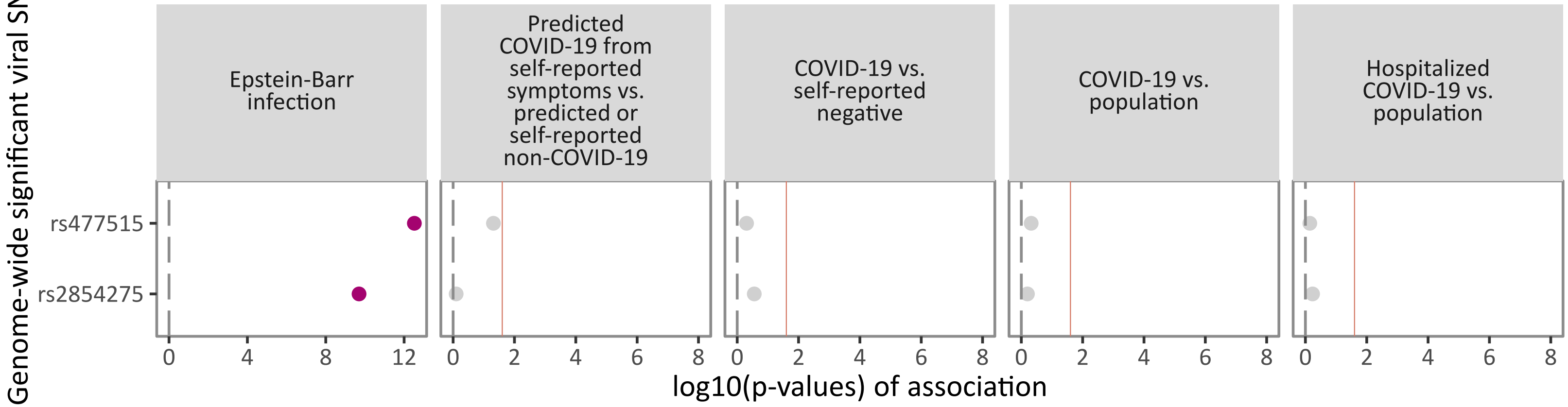

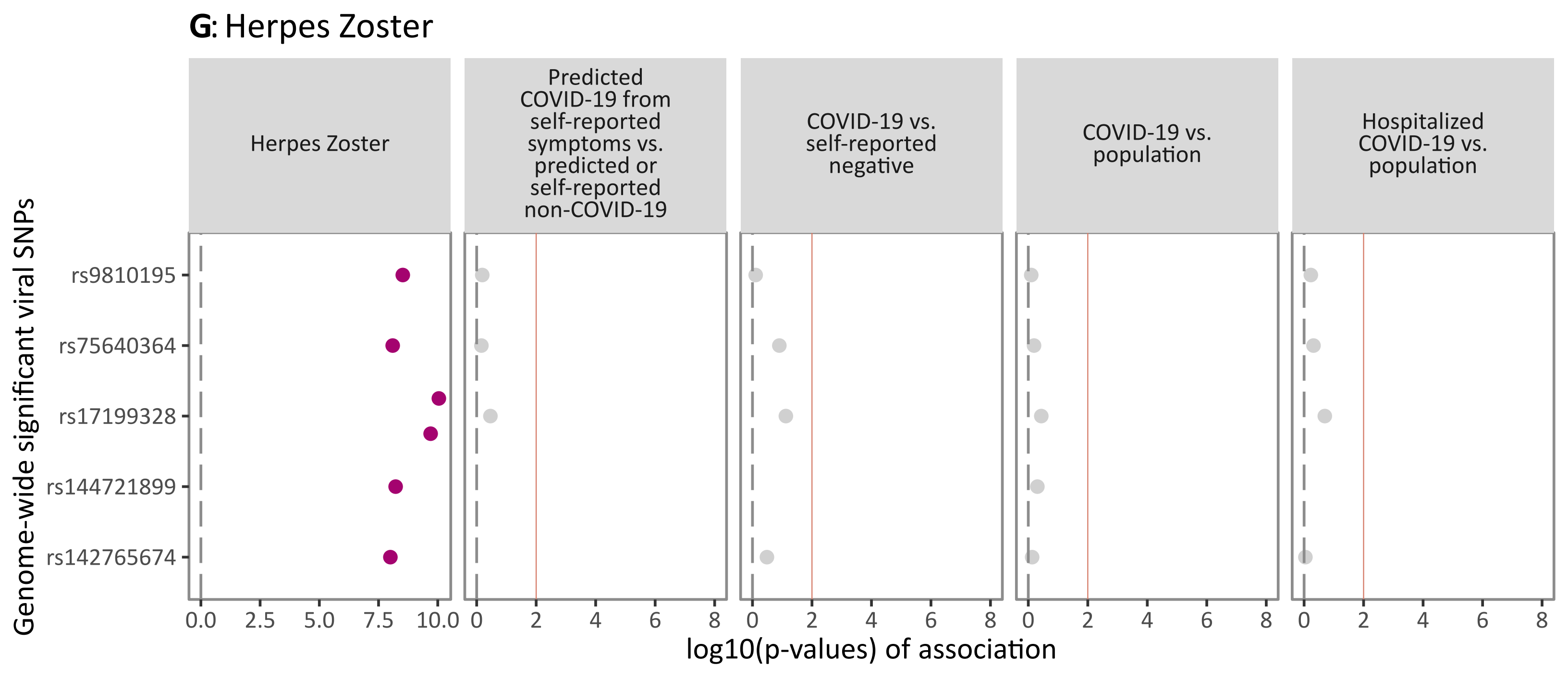

G: Herpes Zoster
Herpes Zoster
Predicted COVID-19 from self-reported symptoms vs. predicted or self-reported non-COVID-19
COVID-19 vs. self-reported negative
COVID-19 vs. population
Hospitalized COVID-19 vs. population
Genome-wide significant viral SNPs
rs9810195
rs75640364
rs17199328
rs144721899
rs142765674
0.0
2.5
5.0
7.5
10.0
0
2
4
6
8
log10(p-values) of association

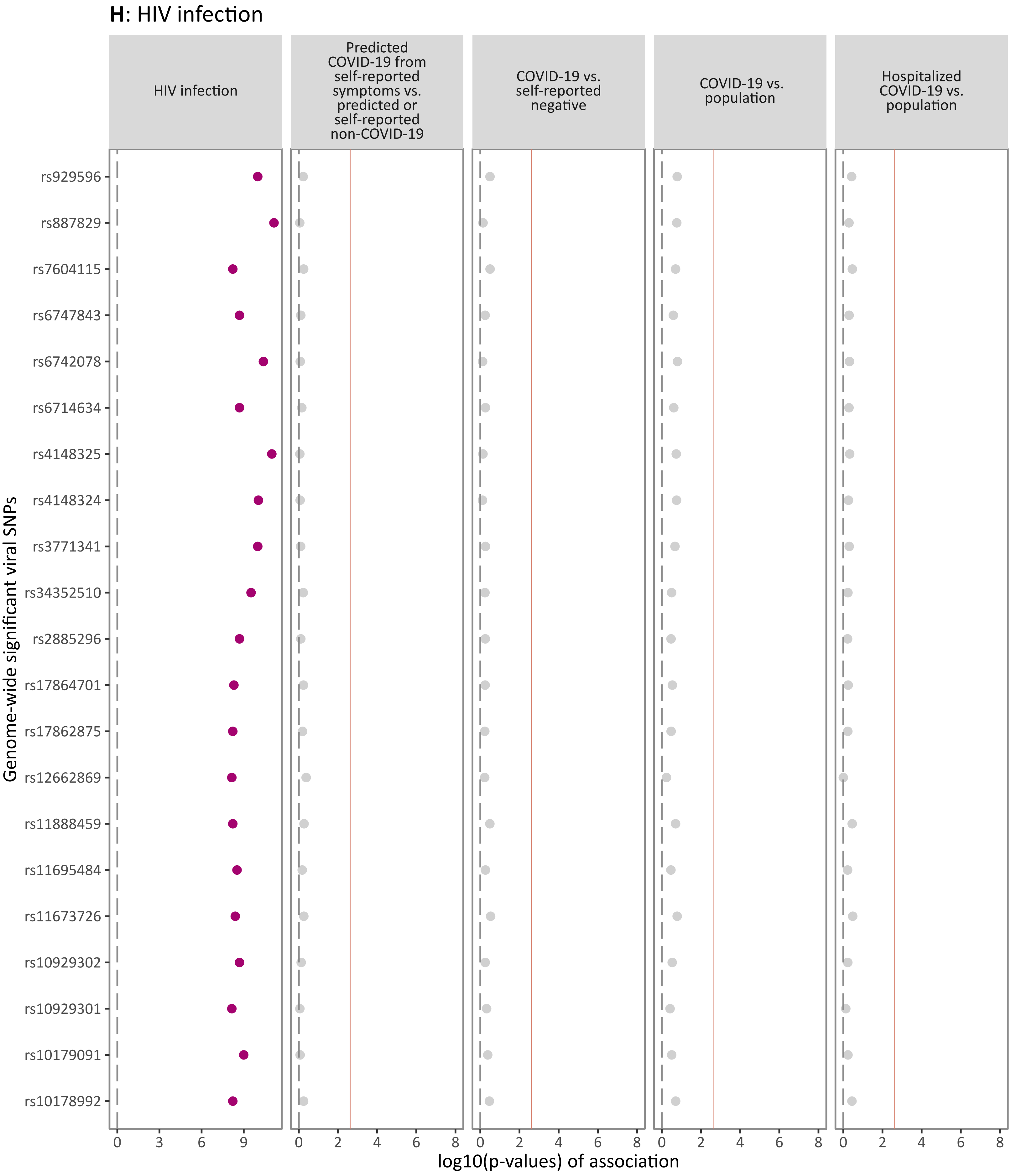
H: HIV infection
HIV infection
Predicted COVID-19 from self-reported symptoms vs. predicted or self-reported non-COVID-19
COVID-19 vs. self-reported negative
COVID-19 vs. population
Hospitalized COVID-19 vs. population
Genome-wide significant viral SNPs
rs929596
rs887829
rs7604115
rs6747843
rs6742078
rs6714634
rs4148325
rs4148324
rs3771341
rs34352510
rs2885296
rs17864701
rs17862875
rs12662869
rs11888459
rs11695484
rs11673726
rs10929302
rs10929301
rs10179091
rs10178992
0 3 6 9
0 2 4 6 8
log10(p-values) of association

I: HIV-1 infection

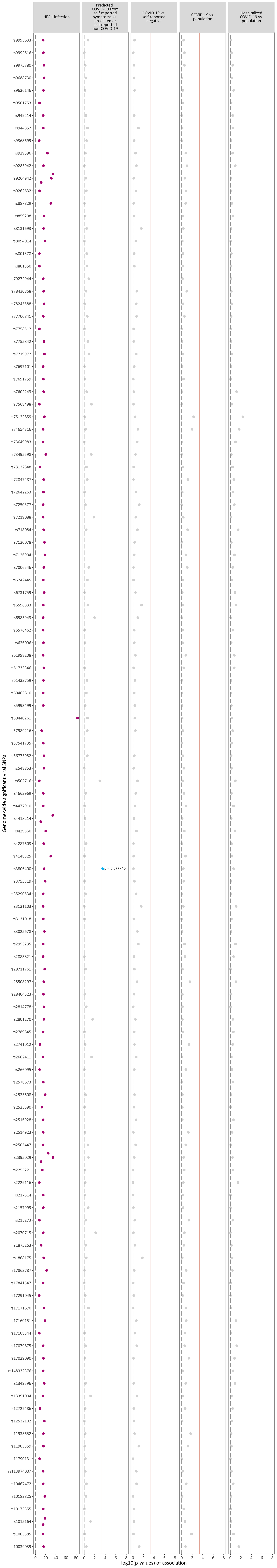

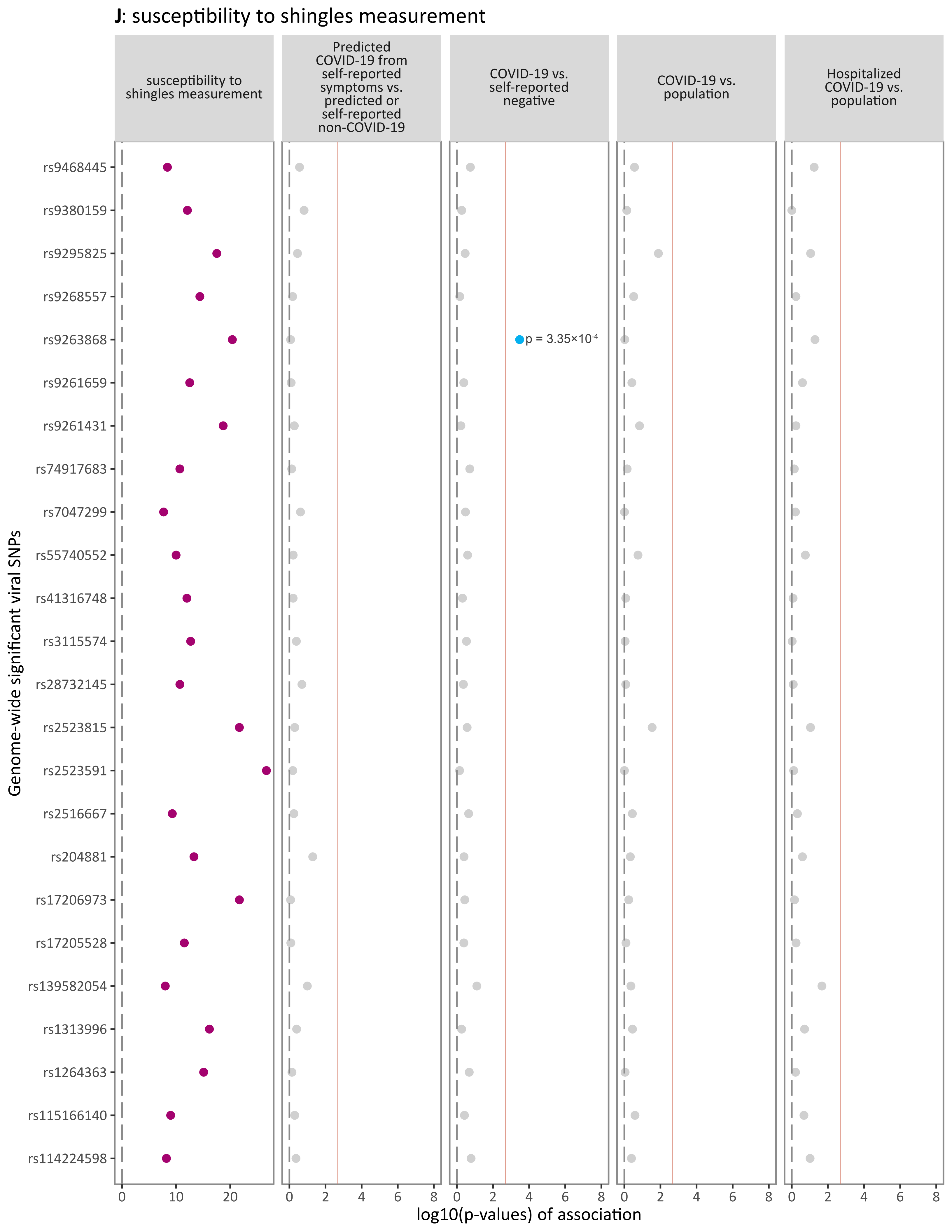
J: susceptibility to shingles measurement
susceptibility to shingles measurement
Predicted COVID-19 from self-reported symptoms vs. predicted or self-reported non-COVID-19
COVID-19 vs. self-reported negative
COVID-19 vs. population
Hospitalized COVID-19 vs. population
rs9468445
rs9380159
rs9295825
rs9268557
rs9263868
rs9261659
rs9261431
rs74917683
rs7047299
rs55740552
rs41316748
rs3115574
rs28732145
rs2523815
rs2523591
rs2516667
rs204881
rs17206973
rs17205528
rs139582054
rs1313996
rs1264363
rs115166140
rs114224598
p = 3.35×10-4
Genome-wide significant viral SNPs
0 10 20
0 2 4 6 8
log10(p-values) of association

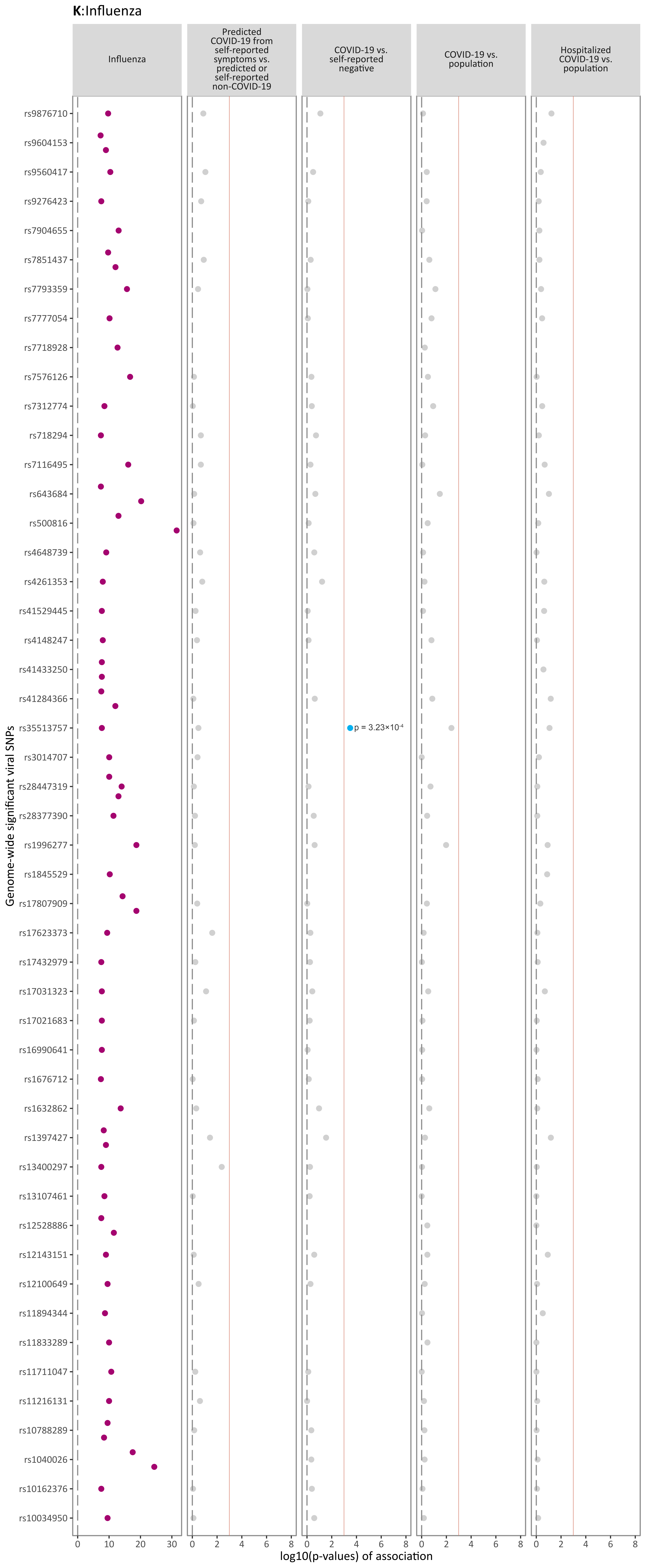

K:Influenza
Influenza
Predicted COVID-19 from self-reported symptoms vs. predicted or self-reported non-COVID-19
COVID-19 vs. self-reported negative
COVID-19 vs. population
Hospitalized COVID-19 vs. population
Genome-wide significant viral SNPs
rs9876710
rs9604153
rs9560417
rs9276423
rs7904655
rs7851437
rs7793359
rs7777054
rs7718928
rs7576126
rs7312774
rs718294
rs7116495
rs643684
rs500816
rs4648739
rs4261353
rs41529445
rs4148247
rs41433250
rs41284366
rs35513757
rs3014707
rs28447319
rs28377390
rs1996277
rs1845529
rs17807909
rs17623373
rs17432979
rs17031323
rs17021683
rs16990641
rs1676712
rs1632862
rs1397427
rs13400297
rs13107461
rs12528886
rs12143151
rs12100649
rs11894344
rs11833289
rs11711047
rs11216131
rs10788289
rs1040026
rs10162376
rs10034950
p = 3.23×10⁻⁴
0 10 20 30
0 2 4 6 8
log10(p-values) of association

L: Susceptibility to mumps measurement

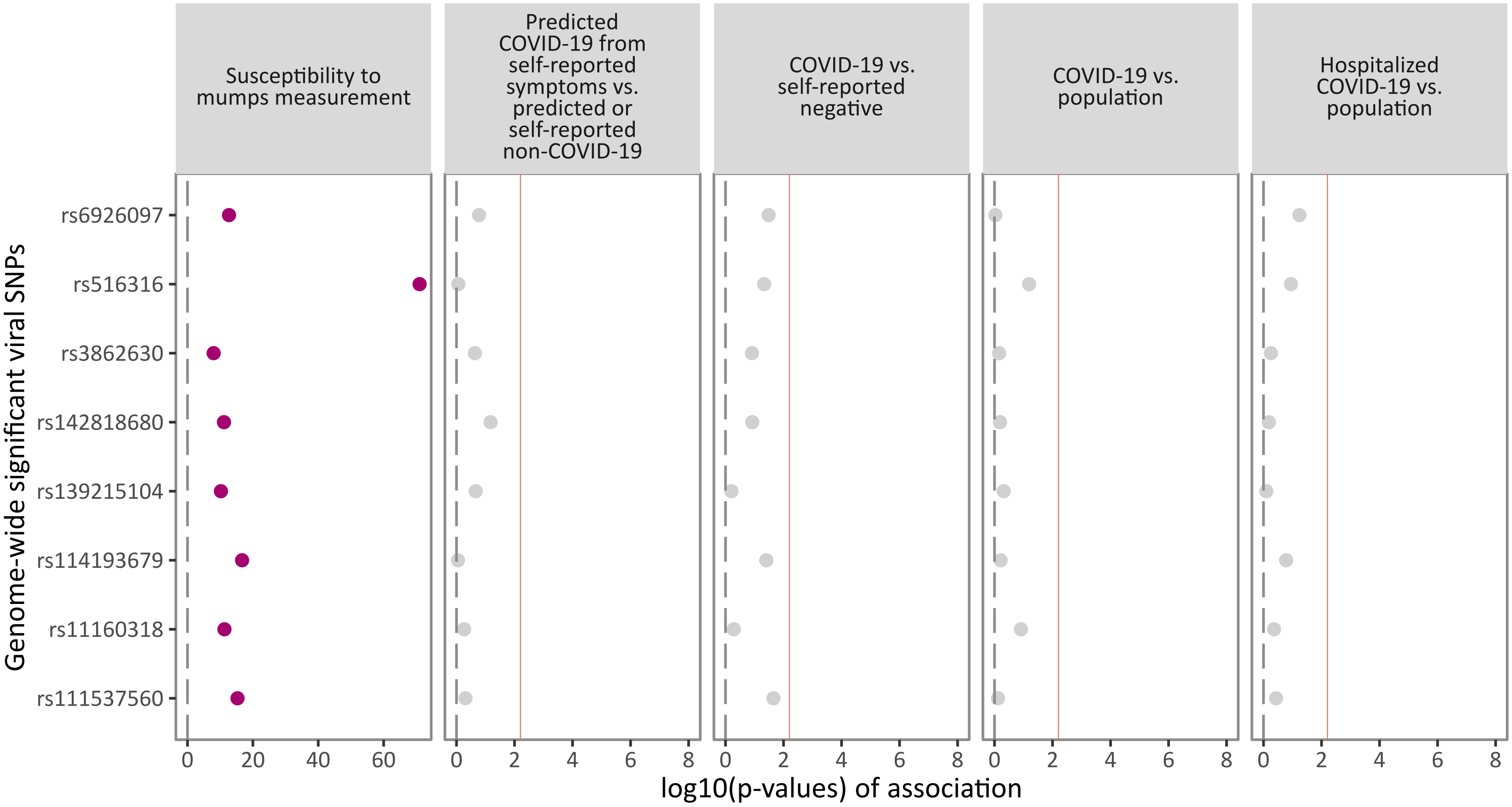

Supplement: S2 Fig — In each of the 5 GWASs in which we replicated an association in a COVID-19 phenotype Europeans constitute the largest portion of included samples. Three of these contained only European samples. The majority of the samples in the COVID-19 analyses are also of European descent. This suggests that these lead SNPs can be compared, and that replication was not caused by ethnicity differences. (PDF) [file pone.0255402.s002.pdf]
