# Supplementary material for: Using symptom-based case predictions to identify host genetic factors that contribute to COVID-19 susceptibility
Source: PLoS One. 2021 Aug 11;16(8):e0255402. doi: 10.1371/journal.pone.0255402 (PMC8357137; doi:10.1371/journal.pone.0255402)
Supplement: S3 Fig — For every COVID-19 GWAS, a genomic inflation factor (λ) with accompanying p-value is shown. Based on both these values and the Q-Q plots, there is no indication of an increased viral infection signal in one of the COVID-19 phenotypes. (PDF) [file pone.0255402.s003.pdf]

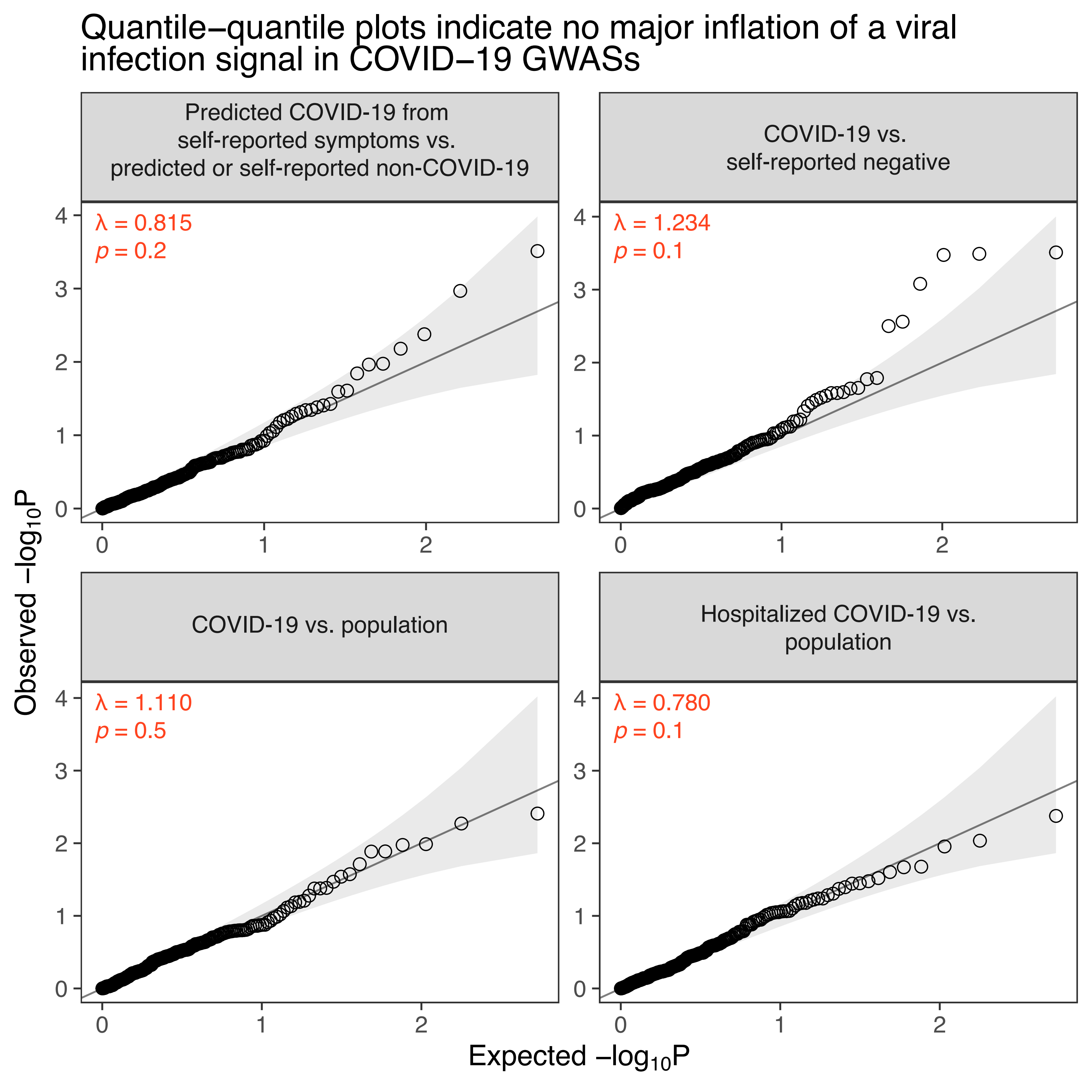

Quantile−quantile plots indicate no major inflation of a viral infection signal in COVID−19 GWASs
Predicted COVID-19 from self-reported symptoms vs. predicted or self-reported non-COVID-19
λ = 0.815
p = 0.2
COVID-19 vs. self-reported negative
λ = 1.234
p = 0.1
COVID-19 vs. population
λ = 1.110
p = 0.5
Hospitalized COVID-19 vs. population
λ = 0.780
p = 0.1
Observed −log10P
Expected −log10P
0
1
2
3
4
