# Supplementary material for: Using symptom-based case predictions to identify host genetic factors that contribute to COVID-19 susceptibility
Source: PLoS One. 2021 Aug 11;16(8):e0255402. doi: 10.1371/journal.pone.0255402 (PMC8357137; doi:10.1371/journal.pone.0255402)
Supplement: S1 Table — (DOCX) [file pone.0255402.s005.docx]

**Table S1**. The different cut-offs and symptoms that were used by the Generation Scotland, Helix, Lifelines and NTR cohorts when applying the Menni COVID-19 prediction model to the datasets prior to running the GWAS.

| **Symptom in Menni model** | **Generation Scotland** | **Helix** | **Lifelines** | **NTR** |
| --- | --- | --- | --- | --- |
| **Loss of smell and taste** | Binary | Binary | 5-point, ≥ 3 | 5-point, ≥ 2 |
| **Severe or significant persistent cough** | Dry cough, binary | Dry cough lasting at least 20 days, binary | Maximum value of cough with and cough without sputum, 5-point, ≥ 3 | Any cough, 5-point, ≥ 4 |
| **Severe fatigue** | Fatigue/ tiredness, binary | Severe fatigue, such as inability to get out of bed, Binary | Minimum of feeling tired, feeling tired quickly, and feeling physically exhausted, left-anchored 7-point, ≤ 2 | Feeling tired or limp,  5-point, > 4 |
| **Skipped meals** | Lack of appetite, binary | Decrease in appetite, Binary | NA | Loss of appetite, 5-point,  ≥ 4 |
