# Supplementary material for: Using symptom-based case predictions to identify host genetic factors that contribute to COVID-19 susceptibility
Source: PLoS One. 2021 Aug 11;16(8):e0255402. doi: 10.1371/journal.pone.0255402 (PMC8357137; doi:10.1371/journal.pone.0255402)
Supplement: S2 Table — (DOCX) [file pone.0255402.s006.docx]

**Table S2.** Full phenotype description and detailed analysis plan used within the C19HG for genome-wide association analysis

**27 April 2020 - version 2.0**

For V1.0 of this document, used for first two rounds of GWAS meta-analyses, please view [her](https://docs.google.com/document/d/1eMdzhO5xk-MACxjz-kOUJLP6Jort5KuwoOa_u-aZPHs/edit?usp=sharing)​ [e](https://docs.google.com/document/d/1eMdzhO5xk-MACxjz-kOUJLP6Jort5KuwoOa_u-aZPHs/edit?usp=sharing)

**Working group**

**Lea Davis,** expertise: genetic medicine, human genomics, EHR based genomics​

**Andrea Ganna,** expertise: statistical genetics, epidemiology​

**Sulggi Lee,** expertise: infectious disease physician, covid research, enrolling a cohort​

**James Priest,** expertise: paed cardiologist, GWAS, health informatics.​

**Alessandra Renieri,** expertise: prof of medical genetics, WES, consultant physician (rare​ diseases), CAT member of EMA.

**Vijay Sankaran,** expertise: paed heamatol/oncol, human geneticist, blood cell traits​

**David van Heel,** expertise: prof of human genetics (Genes & Health cohort CI), consultant​ physician (adults) Barts Health NHS Trust, deputy Chief Clinical Information Officer, clinical IT on covid patients (e.g. for Nightingale & Barts Health).

**Patrick Deelen**, expertise: postdoc Lifelines (Lude Franke), bioinformatician human genetics​

**Brent Richards**, expertise: prof of medicine, endocrinology, seeing covid patients, genetic​ epidemiology.

**Tomoko Nakanishi:** respiratory doctor, PhD.​

**Les Biesecker**, paed doctor, medical geneticist, PI covid cohort US NIH (recruiting)​

**Eric Kerchberger**:​ pulmonary clinical fellow, MD. Critical illness syndromes.

**RATIONALE:** ​​Identification of human genetic factors (should these exist) impacting the COVID19 pandemic holds the potential to rapidly offer important information. Within individual cohorts, we propose establishing a shared set of phenotypes to be combined with genomic data for standard GWAS and further meta-analysis by the analysis group. Analyses will be simple and scalable.


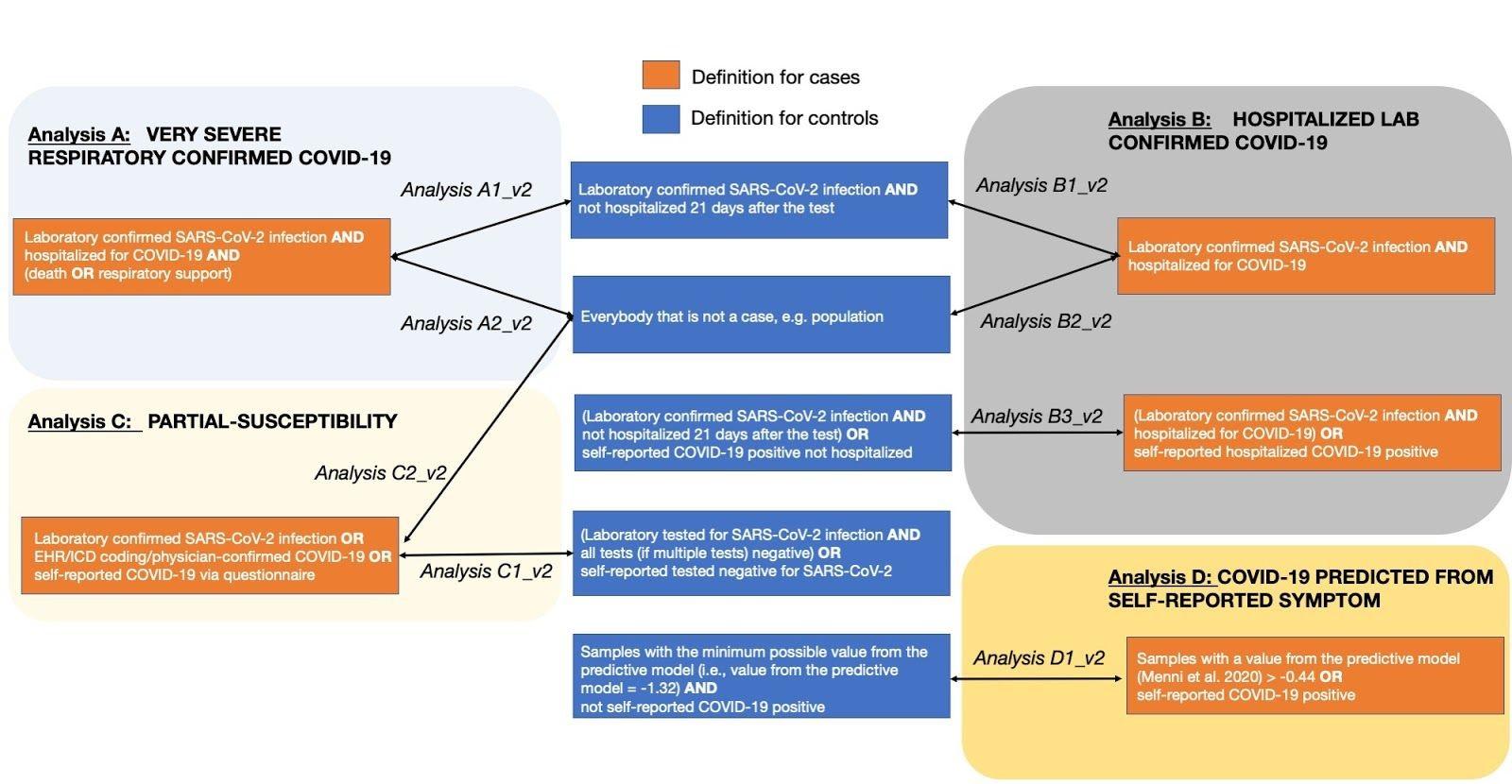


**Analysis A:** ​ ​**VERY**​ ​**SEVERE RESPIRATORY CONFIRMED COVID**

Analysis A1_v2 ​(name ANA_A1_V2) ​-- PRIMARY ANALYSIS

**Cases:**​ ​Hospitalized laboratory confirmed​ SARS-CoV-2 infection (RNA and/or serology based), AND (​death​ OR ​respiratory support​ (intubation, CPAP, BiPAP, CNP (continue external negative pressure), Optiflow/very high flow Positive End Expiratory Pressure Oxygen* - (see ​**Appendix 1** for suggestive codes) AND hospitalization with COVID19 as primary reason for admission..

*Simple supplementary oxygen (e.g. 2 litres/minute via nasal cannulae) ​only​ does not qualify for case status.

**Controls:** ​Laboratory confirmed SARS-CoV-2 infection (RNA and/or serology based) AND not hospitalised 21 days after the test.

**Analysis A2_v2**​ ​(name ANA_A2_V2)

**Cases:**​ As A1_v2

**Controls:** ​everybody that is not a case, e.g. population

**Analysis B:** ​**HOSPITALIZED LAB CONFIRMED COVID**

Analysis B1_v2 ​(name ANA_B1_V2) ​-- PRIMARY ANALYSIS

**Cases:**​ ​Hospitalized laboratory confirmed​ SARS-CoV-2 infection (RNA and/or serology based), hospitalization ​**due to corona-related symptoms**​.

**Controls:** ​Laboratory confirmed SARS-CoV-2 infection (RNA and/or serology based) AND not hospitalised 21 days after the test.

**Analysis B2_v2**​ ​(name ANA_B2_V2)

**Cases:**​ As B1_v2

**Controls:** ​everybody that is not a case, e.g. population

**Analysis B3_v2** ​(name ANA_B3_V2)

**Cases:**​ ​Hospitalized laboratory confirmed​ SARS-CoV-2 infection (RNA and/or serology based), hospitalization due to corona-related symptoms OR ​self-reported hospitalized COVID-19 positive​ (e.g. by questionnaire)

**Controls:** ​(Laboratory confirmed SARS-CoV-2 infection (RNA and/or serology based) AND not hospitalised 21 days after the test) OR ​self-reported COVID-19 positive without hospitalization **Analysis C:** ​**PARTIAL**​-​**SUSCEPTIBILITY**

**Analysis C1_v2** ​(name ANA_C1_V2)

**Cases:**​ individuals with ​laboratory confirmation​ of SARS-CoV-2 infection (RNA and/or serology based) OR ​EHR/ICD coding/ Physician Confirmed​ COVID-19 (See ​**Appendix 1**​ for suggestive codes) OR ​self-reported COVID-19 positive​ (e.g. by questionnaire)

**Controls:** ​(Laboratory tested for SARS-CoV-2 infection (RNA and/or serology based) AND all tests (if multiple tests) ​negative​*) OR ​self-reported​ tested negative for SARS-CoV-2 infection (e.g. by questionnaire)

*​We suggest to exclude from the control group individuals with high clinical suspicion of COVID-19 but negative laboratory tests

# Analysis C2_v2​ ​(name ANA_C2_V2)​ -- PRIMARY ANALYSIS

**Cases:** ​As C1_v2

**Controls:** ​everybody that is not a case, e.g. population

**Analysis D: COVID-19 PREDICTED FROM SELF-REPORTED SYMPTOMS**

**Analysis D1_v2 (**name ANA_D1_V2​ **)**​

**Cases:** Samples with a value from the predictive model* > -0.44 OR ​ self-reported COVID-19​ positive

*The model from ​[Menni et al. 2020](https://www.nature.com/articles/s41591-020-0916-2)​, minus age and sex predictors, will be applied to self-report data. The published model is as follows:

Predictionmodel=−1.32+(1.75×lossofsmellandtaste)+(0.31×severeorsignificantpersistentcough)+(0.49×severefatigue)

+(0.39×skippedmeals) where all symptoms are coded as 1 if the person self-reports the symptom and 0 if not.

The AUC for the original version of this model, which included age and sex, has been shown in both the Helix and Lifelines dataset to be 0.79 despite slight differences in the phenotypes (for example, skipped meals were not included in the Lifelines model, and Helix used any dry cough lasting at least 20 days as a predictor instead of self-reported severe or persistant cough). In the Helix dataset, we have observed that removing age and sex from the model, which makes more sense for genetic analyses, results in minimal changes of the AUC (to 0.78). In the Helix dataset, the final model produces PPV of 0.41, sensitivity 0.62, and specificity 0.88.

**Controls:** Samples with the minimum possible value from the predictive model (i.e., value from​ the predictive model = -1.32) AND NOT self-reported COVID-19 positive

Please note that **age, age^2, sex and age*sex are included as a covariate in all analyses**​  **A,B,C,D above. Therefore these analyses already consider, to some extent, the difference between younger and older (with e.g. more comorbidities) patients.**

# ANALYSIS PHENOTYPES FOR CONSIDERATION IN V3

Mortality in young individuals

Min lymphocyte levels during hospitalization

Max granulocyte and monocyte levels during hospitalization

Max IL-6 levels during hospitalization

Max CRP levels during hospitalization

Min Fibrinogen levels during hospitalization

Severity scale during hospitalization

Loss of smell or taste

Neurological comorbidities (encephalitis and meningitis).

Max Blood lactate levels as indicator of shock

Min Ferrtin Level

Coagulation Profile ( PT , APTT , TT , Platelet count , D-dimer , Plasma fibrinogen) Hypercoagulation (D-dimers, lupus anticoagulant, cardiolipin antibodies and anti-B2G1 antibodies)

# APPENDIX 1:​

Diagnostic codes for COVID-19 severity (provided by Lea Davis and Julia Sealock) <https://drive.google.com/file/d/1ck0ABYZ6oYnMStoYnGpnA7n1W6wcY3_6/view>
