# Supplementary material for: Using symptom-based case predictions to identify host genetic factors that contribute to COVID-19 susceptibility
Source: PLoS One. 2021 Aug 11;16(8):e0255402. doi: 10.1371/journal.pone.0255402 (PMC8357137; doi:10.1371/journal.pone.0255402)
Supplement: S3 Table — Due to absence of testing data, Generation Scotland could not be used for replication of the Menni COVID-19 prediction model and in the development of the Lifelines COVID-19 prediction model. (DOCX) [file pone.0255402.s007.docx]

**Table S3.** Descriptive statistics of the Generation Scotland, Helix, Lifelines and NTR cohorts. Due to absence of testing data, Generation Scotland could not be used for replication of the Menni COVID-19 prediction model and in the development of the Lifelines COVID-19 prediction model.

|  | **Helix** | | **Lifelines** | | **NTR** | |
| --- | --- | --- | --- | --- | --- | --- |
|  | Positive test | Negative test | Positive test | Negative test | Positive test | Negative test |
| N (%) | 27 (12.5) | 189 (87.5) | 56 (8.7) | 586 (91.3) | 85 (18.2) | 382 (81.8) |
| Age, yr, mean (sd) | 49 (14.4) | 51 (15.3) | 52.4 (9.2) | 52.2 (12.3) | 41.0 (13.1) | 41.3 (12.9) |
| Male sex, N (%) | 11 (40.7) | 58 (30.7) | 14 (25.0) | 155 (26.5) | 10 (13.3) | 65 (86.7) |
| BMI, kg/m^2^, mean (sd) | 28.6 (7.8) | 28.7 (6.4) | 26.2 (4.3) | 26.4 (4.7) | 25.4 (4.6) | 25.4 (4.8) |
| Current smoker, N (%) | 1 (4.0) | 18 (10.0) | 3 (5.4) | 52 (8.9) | 6 (11) | 48 (89) |
