# Supplementary material for: Using symptom-based case predictions to identify host genetic factors that contribute to COVID-19 susceptibility
Source: PLoS One. 2021 Aug 11;16(8):e0255402. doi: 10.1371/journal.pone.0255402 (PMC8357137; doi:10.1371/journal.pone.0255402)
Supplement: S4 Table — Logistic regression for each symptom separately in Lifelines on positive (n = 56) vs negative (n = 586) tested subjects to define symptom cut-offs (reference = absence of symptom). (DOCX) [file pone.0255402.s008.docx]

**Table S4**. Overlapping symptoms in the Helix, Lifelines and NTR cohorts. Logistic regression for each symptom separately in Lifelines on positive (n=56) vs negative (n=586) tested subjects to define symptom cut-offs (reference = absence of symptom).

|  | **Low symptom severity** | **Intermediate/high symptom severity** | **Cut-off to define positive symptom** |
| --- | --- | --- | --- |
|  | OR (p) | OR (p) |  |
| Difficulty breathing | 2.57 (0.003) | 2.82 (0.006) | Low |
| Runny nose | 1.01 (0.959) | 2.68 (0.006) | Intermediate/high |
| Sore throat | 1.88 (0.064) | 2.42 (0.014) | Low |
| Cough | 4.31 (0.009) | 9.29 (0.000) | Low |
| Fever | 11.83 (0.000) | 14.66 (0.000) | Low |
| Diarrhea/stomach ache | 1.87 (0.048) | 2.43 (0.018) | Low |
| Loss taste/smell | 2.47 (0.047) | 21.03 (0.000) | Intermediate/high |
| Fatigue | 1.85 (0.306) | 7.22 (0.000) | Intermediate/high |
