# Supplementary material for: Using symptom-based case predictions to identify host genetic factors that contribute to COVID-19 susceptibility
Source: PLoS One. 2021 Aug 11;16(8):e0255402. doi: 10.1371/journal.pone.0255402 (PMC8357137; doi:10.1371/journal.pone.0255402)
Supplement: S5 Table — The Lifelines COVID-19 prediction model (a). Diagnostics of different cut-offs of predicted probability of the Lifelines COVID-19 prediction model (b). Model diagnostics of the Lifelines COVID-19 prediction model in the Helix, Lifelines and NTR cohorts (c). (DOCX) [file pone.0255402.s009.docx]

**Table S5a**. The Lifelines COVID-19 prediction model

|  | **B** | **SE** | **p** | **OR** | **OR 95% CI** |
| --- | --- | --- | --- | --- | --- |
| Constant | -4.497 | 0.541 | 0.000 | 0.011 |  |
| Cough | 1.032 | 0.560 | 0.065 | 2.807 | 0.938 - 8.406 |
| Fever | 2.042 | 0.343 | 0.000 | 7.709 | 3.934 - 15.106 |
| Loss of smell or taste | 2.145 | 0.363 | 0.000 | 8.545 | 4.197 - 17.397 |

**Table S5b**. Diagnostics of different cut-offs of predicted probability of the Lifelines COVID-19 prediction model

| **Cut-off predicted probability** | **Sensitivity** | **Specificity** | **Positive predicted value** | **Negative predicted value** |
| --- | --- | --- | --- | --- |
| > 0.10 | 0.821 | 0.848 | 0.341 | 0.980 |
| > 0.20 | 0.500 | 0.952 | 0.500 | 0.952 |
| > 0.30 | 0.357 | 0.974 | 0.571 | 0.941 |
| > 0.40 * | 0.357 | 0.974 | 0.571 | 0.941 |
| > 0.50 | 0.339 | 0.974 | 0.559 | 0.939 |

* A predicted probability > 0.4 was used to define a positive predicted case.

**Table S5c**. Model diagnostics of the Lifelines COVID-19 prediction model in the Helix, Lifelines and NTR cohorts.

| **Cohort** | **AUC (95% CI)** | **Sensitivity** | **Specificity** | **Positive predicted value** | **Negative predicted value** |
| --- | --- | --- | --- | --- | --- |
| Helix | 0.742 (0.686-0.869) | 0.333 | 0.943 | 0.444 | 0.912 |
| Lifelines | 0.882 (0.834-0.930) | 0.357 | 0.974 | 0.571 | 0.941 |
| NTR | 0.848 (0.801-0.895) | 0.422 | 0.958 | 0.700 | 0.878 |

The model: -4.497 + 1.032*cough + 2.042*fever + 2.145*loss of smell or taste. A predicted probability cut-off of > 0.40 is used to define a positive predicted case.
