# Supplementary material for: Using symptom-based case predictions to identify host genetic factors that contribute to COVID-19 susceptibility
Source: PLoS One. 2021 Aug 11;16(8):e0255402. doi: 10.1371/journal.pone.0255402 (PMC8357137; doi:10.1371/journal.pone.0255402)
Supplement: S1 Results — (DOCX) [file pone.0255402.s011.docx]

*Prevalence of core predicted COVID-19 symptoms in Lifelines*

In the Lifelines COVID-19 cohort, we observe that individuals who were tested for SARS-CoV-2 more often report working in essential occupations, for example in healthcare, and were generally younger. Individuals who tested positive for an infection report having been in contact with another infected individual (62.5%) more often than individuals with a negative test outcome (19.3%) (**Table SR1**). Furthermore, infected individuals were already reporting symptoms of fever, loss of smell or taste, fatigue and coughing at higher frequencies even before testing positive and continued to report these symptoms thereafter (**Figure SR1**). Individuals with a negative test outcome reported few to no symptoms of fever and loss of smell or taste, overall. Individuals predicted for potential COVID-19 show a pattern of reported symptoms more similar to that of individuals with a positive test outcome. While, as expected, the prevalence of loss of smell or taste, fatigue and coughing is highest at the time of the positive test, these symptoms are also still reported afterwards. For predicted COVID-19 cases, fewer symptoms of fever are reported than for cases with a positive test outcome.

**Table SR1.** Reported number of individuals with an essential occupation and who have been in contact with infected individuals across negative, positive, and predicted COVID-19 cases in the Lifelines cohort.

| **Lifelines COVID-19 cohort** | **Works in an essential occupation (%)** | **Been in contact with an individual with a confirmed SARS-CoV-2 infection (%)** |
| --- | --- | --- |
| Negative test | 358 (61.1) | 113 (19.3) |
| Positive test | 43 (76.8) | 35 (62.5) |


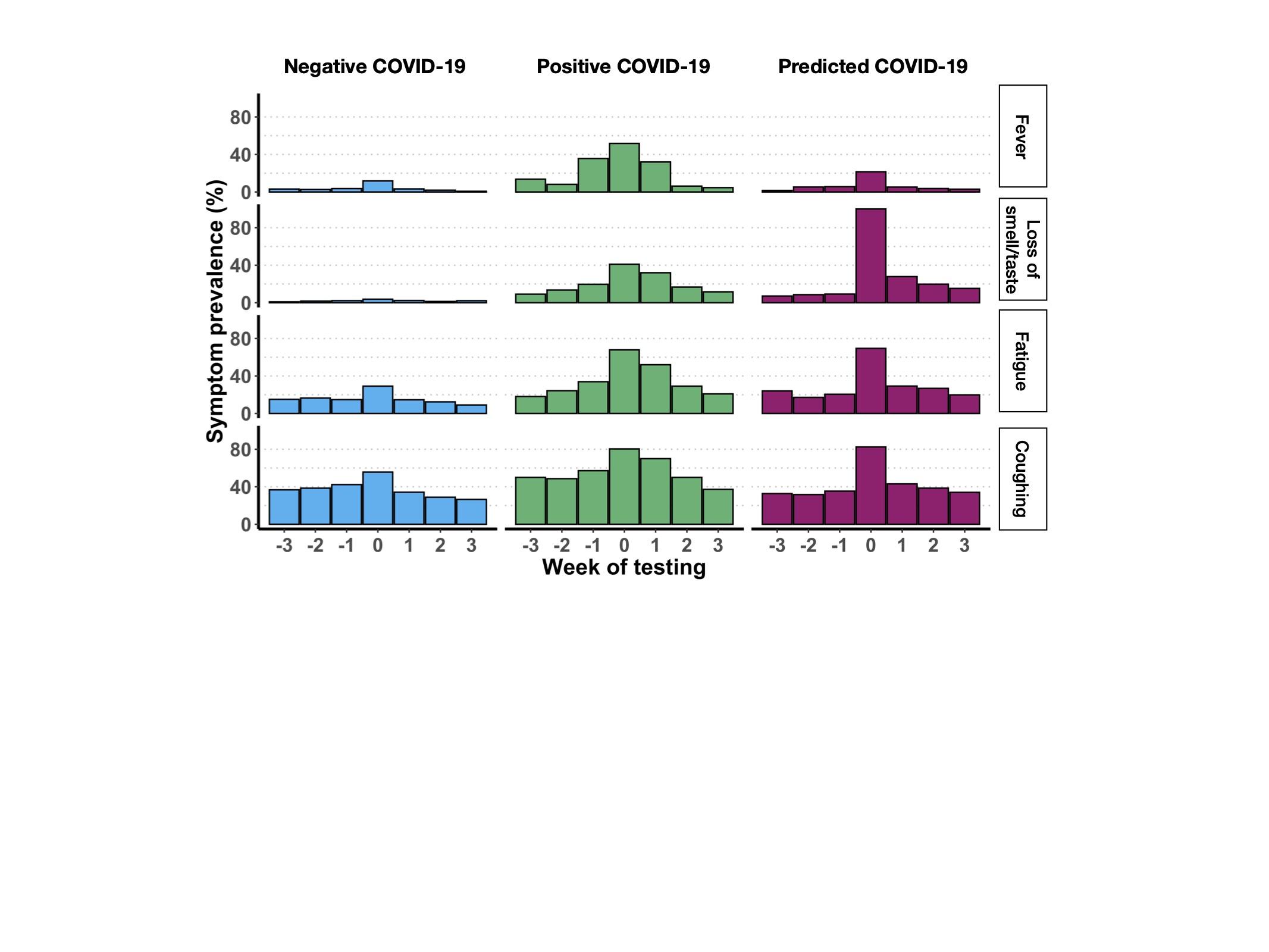
**Figure SR1**. Prevalence of self-reported symptoms before and after testing negative, testing positive and predicted COVID-19 in the Lifelines cohort. To compare self-reported symptoms between cases with a negative test outcome (blue), a positive test outcome (green) and a predicted case status (purple), we aligned weekly reported symptoms for each individual to the week of testing (i.e. week 0). For each week, symptom prevalence (y-axis) was calculated and visualized in relation to the week of testing (x-axis). Shown are symptoms used as input in the Menni COVID-19 prediction model and fever.

*Phenotypic associations between predicted COVID-19 and co-morbidities in Lifelines*

To gain insights into disease associations within the group of predicted COVID-19 cases, we explored if predicted cases report specific pre-existing conditions more often than the controls in our GWAS. Here we observe a positive association with self-reported lung disease, chronic muscle disease, psychiatric illness, cancer and neurological disease (**Figure S4**). This was not observed when comparing patients with a positive versus negative test outcome. While this may indicate that predicted COVID-19 cases present a different profile of pre-existing conditions as compared to true cases, the comparison between predicted and positive COVID-19 cases remains biased due to testing policies and the small number of cases with a positive test outcome.
