# Supplementary material for: Using symptom-based case predictions to identify host genetic factors that contribute to COVID-19 susceptibility
Source: PLoS One. 2021 Aug 11;16(8):e0255402. doi: 10.1371/journal.pone.0255402 (PMC8357137; doi:10.1371/journal.pone.0255402)
Supplement: S1 Methods — (DOCX) [file pone.0255402.s012.docx]

**Supplementary Methods**

*Cohort-level GWAS in Generation Scotland*

Generation Scotland samples were genotyped using Illumina arrays HumanOmniExpressExome8v1-2_A or HumanOmniExpressExome-8v1_A. This was followed by phasing using Shapeit v2.r873 + duohmm and imputation using the HRC.r1-1 panel. A GWAS of the subset samples available for COVID-19 analysis was performed using SAIGE v.0.35.8.8. Individuals with a predicted COVID-19 score above -0.44 were considered a case, and individuals with the minimum possible score (-1.32) were considered a control. Age and sex were excluded from the model for the GWAS. Age, sex, age * sex, age * age and the first 20 principal components were included in the association analysis.

*Cohort-level GWAS in Helix*

Helix samples underwent Exome+ sequencing, as previously described (1). This includes exome sequencing plus a microarray-equivalent SNP backbone. The resulting data were processed using a custom version of Sentieon and aligned to GRCh38, with variant calling and phasing algorithms following GATK best practices (2). For imputation of common variants, pre-phasing was performed using reference databases, which include the 1000 Genomes Phase 3 data. This was followed by genotype imputation for all 1000 Genomes Phase 3 sites that have genotype quality values less than 20. Imputation results were then filtered for quality so that only high precision imputed variant calls were reported.

A GWAS of common and low frequency variants was performed using SAIGE v.39. Individuals with a predicted COVID-19 score above -0.44 were considered a case, and individuals with the minimum possible score (-1.32) were considered a control. Age and sex were excluded from the model for the GWAS. A representative, genome-wide set of 184,445 LD-pruned, high-quality common variants was used to both build principal components and build the analysis model, as previously described (3). Age, sex, age * sex, age * age, age * age * sex, the first 10 principal components and the Helix bioinformatics pipeline version were included as covariates in the model.

*Cohort-level GWAS in Lifelines*

Genotyping was done on two platforms over time, namely HumanCytoSNP and Infinium Global Screening Array® (GSA). On each platform, genotyping was performed following manufacturer’s instructions.

The first subset of samples were genotyped with two different versions of HumanCytoSNP (12v2.0 and 12v2.1). The versions were harmonized and unified by using only probes that were present in both platforms, mapping to the same genome build (37), and making sure that resulting location was concordant with either 1000 Genomes phase 3 or Genome of the Netherlands (GoNL) data. Following this, the genotype calling quality control included removing samples with any of the following: call rate <0.98, heterozygosity > 4 SD above the mean for autosomes, mismatching sex genotypes (heterozygosity of X chromosome > 0.005 for males, and < 0.1 for females), and ethnic outliers (PC >2 SD from mean PC, for the first 10 PCs); and excluding variants with deviations from Hardy-Weinberg equilibrium (p < 1x10^-6^) or call rate < 0.015. Finally, the genotypes were phased with SHAPEIT2 r790.

The other subset of samples were genotyped using the Infinium Global Screening Array® (GSA) MultiEthnic Disease Version. We performed quality controls on both samples and markers. A detailed description has previously been described[^23^](https://www.zotero.org/google-docs/?JypyzH). In brief, quality control included removal of samples and variants with a low genotyping call rate (<99%), variants showing deviation from Hardy-Weinberg equilibrium (p < 1 × 10^−6^) or excess of Mendelian errors in families (>1% of the parent-offspring pairs), and samples with very high or low heterozygosity. We removed samples that did not show consistent information between reported sex and genotypes on the X chromosome, between reported familial information and observed identity-by-descent sharing with family members.

Both datasets mentioned above were imputed using the Haplotype Reference Consortium (HRC) panel v1.1 at the Sanger imputation server1(4).

To perform a GWAS for predicted COVID-19 case-control status, we applied the Menni model as described in previous sections excluding age and sex as prescribed in the C19HG analysis plan. Individuals with a predicted COVID-19 score above -0.44 or a self-reported positive test were considered a case, and individuals with the minimum possible score (-1.32) without a self-reported positive test were considered a control. Participants were excluded from analyses if they never answered any of the questions about their COVID-19 status (regarding self-diagnosis, diagnosis by a physician, or testing status) in one of the first 7 questionnaires, or if the predicted COVID-19 score could not be calculated. After having determined case-control status of individuals, we performed the genome-wide association analysis using SAIGEgds (5). SAIGEgds, based on SAIGE (6), is an R-package with an implementation of a generalized mixed model. It accounts for sample relatedness and case-control imbalance.

In using SAIGEgds (version 1.0.2), two steps were performed. In the first step, the null logistic mixed model was fitted to estimate the variance component, the effect of the parameters age, age^2^ and sex, and interaction effects between age and sex. In the second step, associations tests between each genetic variant and predicted COVID-19 case-control status were performed. We applied a minimum minor allele frequency (MAF) threshold of 1%.

*Cohort-level GWAS in NTR*

Genotyping was done on multiple platforms over time, namely Perlegen-Affymetrix, Affymetrix 6.0, Affymetrix Axiom, Illumina Human Quad Bead 660, Illumina Omni 1M and Illumina GSA. On each platform, genotyping was performed following manufacturers protocols, using the then appropriate calling software. For each genotype platform, samples were removed if DNA sex did not match the expected phenotype, if the PLINK heterozygosity F statistic was < -0.10 or > 0.10, or if the genotyping call rate was < 0.90. SNPs were removed if the MAF < 1×10-6, if the Hardy-Weinberg equilibrium p-value was < 1×10-6, and/or if the call rate was < 0.95. Subsequently, for each platform, the genotype data was aligned with the 1000 Genomes reference panel using the HRC and 1000 Genomes checking tool, which tests and filters for SNPs with allele frequency differences larger than 0.20 as compared to the CEU population, palindromic SNPs and DNA strand issues. The data of the six platforms was then merged into a single dataset, keeping all quality controlled SNPs of each platform. For each individual, one platform was chosen. Based on the ~10.8k SNPs that all platforms have in common, DNA Identity By Descent state was estimated for all individual pairs using the Plink and King programs. These estimates were then compared to the expected familial relations, and samples were removed if these failed to fit. CEU population outliers, based on per platform 1000 Genomes projection with the Smartpca software, were removed from the data. Then, per platform, the data was phased using Eagle and then imputed to 1000 Genomes and Topmed using Minimac. Post imputation, the resulting separate platform Variant Call Format (VCF) files were merged with Bcftools into a single VCF file per chromosome for each reference, only for those SNPs present on all six platforms. These data were then converted to BGEN genotypes using Bgenix, and these were used in the association analyses. Finally, 20 PCs were re-calculated with Smartpca using LD-pruned 1000 Genomes–imputed SNPs that were also genotyped on at least one platform, had MAF > 0.05 and were not present in the long-range LD regions, as described earlier.

After determining case-control status of individuals, we performed the genome-wide association analysis using the SAIGE (7) v0.38 docker package on the Topmed imputed data with a prior R2<0.30 imputation filter. Individuals with a predicted COVID-19 score above -1.32 considered a case and individuals with a minimum possible score (-1.32) considered a control. Two steps were performed. In the first step, taking into account the Genetic Relationship Matrices (GRM) of individuals, the null logistic mixed model was fitted to estimate the variance components and the effect of the following parameters: the 5 genotype platforms (Affymetrix 6 as reference), PC1–PC20, age, age^2^, sex and interaction effects between age and sex. In the second step, the alternative model associations tests between each genetic variant and predicted COVID-19 case-control status were performed. During the analyses, SNPs with a MAF < 0.0001 and a Minimum minor allele count (MAC) < 1 were removed.

*COVID-19 Host Genetics Initiative meta-analysis*

C19HG GWAS meta-analysis round 3 results were used in this study. Each participating cohort submitted GWAS summary stats for one to four of the COVID-19 phenotype definitions. Suggested covariates for each GWAS were sex, age, age*age, sex*age, and 20 principal components. Summary stats of individual studies were manually examined for inflation, deflation, and suspicious number of false positives. Qualifying summary stats were filtered to INFO > 0.6 and MAF > 0.0001 prior to meta-analysis. Meta-analysis was done using inverse variance weighting of effects, accounting for strand differences and allele flips in the individual studies. Meta-analysis results of variants appearing in at least three studies (analysis *C2*) or two studies (all other analyses) were made publicly available. The meta-analysis software and workflow are available at <https://github.com/covid19-hg/META_ANALYSIS>

**References**

1. Helix. Variants Pipeline Performance White Paper [Internet]. 2019 [cited 2020 Aug 18]. Available from: https://cdn.shopify.com/s/files/1/2718/3202/files/Helix_Performance_White_Paper_v4.pdf

2. Kendig KI, Baheti S, Bockol MA, Drucker TM, Hart SN, Heldenbrand JR, et al. Computational performance and accuracy of Sentieon DNASeq variant calling workflow [Internet]. Bioinformatics; 2018 Aug [cited 2020 Aug 18]. Available from: http://biorxiv.org/lookup/doi/10.1101/396325

3. Cirulli ET, White S, Read RW, Elhanan G, Metcalf WJ, Tanudjaja F, et al. Genome-wide rare variant analysis for thousands of phenotypes in over 70,000 exomes from two cohorts. Nat Commun. 2020 Dec;11(1):542.

4. The 1000 Genomes Project Consortium. A global reference for human genetic variation. Nature. 2015 Oct 1;526(7571):68–74.

5. Zheng X, Davis J. SAIGEgds – an efficient statistical tool for large-scale PheWAS with mixed models. 2019.

6. Zhou W, Nielsen JB, Fritsche LG, Dey R, Gabrielsen ME, Wolford BN, et al. Efficiently controlling for case-control imbalance and sample relatedness in large-scale genetic association studies. Nat Genet. 2018 Sep;50(9):1335–41.

7. Mc Intyre K, Lanting P, Deelen P, Wiersma H, Vonk JM, Ori AP, et al. The Lifelines COVID-19 Cohort: a questionnaire-based study to investigate COVID-19 infection and its health and societal impacts in a Dutch population-based cohort. medRxiv. 2020 Jun 24;2020.06.19.20135426.
