# Supplementary material for: Using symptom-based case predictions to identify host genetic factors that contribute to COVID-19 susceptibility
Source: PLoS One. 2021 Aug 11;16(8):e0255402. doi: 10.1371/journal.pone.0255402 (PMC8357137; doi:10.1371/journal.pone.0255402)
Supplement: Attachment — Submitted filename: Response to reviewers_PONE-D-20-38159.docx [file pone.0255402.s013.docx]

**Rebuttal Letter PONE-D-20-38159**

**Title manuscript:** Using symptom-based case predictions to identify host genetic factors that contribute to COVID-19 susceptibility.

**Corresponding author**: Lude H. Franke

We are grateful for the useful feedback of reviewers and thank the editor for the opportunity to revise our manuscript. In a point-by-point manner, we answer the reviewer's comments below and hope that this addresses any remaining concerns for publication in PLOS ONE. Furthermore, we have revised the formatting and provided information on this in the section below the rebuttal letter.

**Editor: The manuscript performed a GWAS analysis to show possible COVID-19 associated SNPs. However, the significance of the results was substantially undermined due to (1) the very limited confirmed positive cases of COVID-19 in the study (n=168), and (2) the low sensitivity of Mennis COVID-19 prediction model. Based on the current available data of COVID-19 cases, a substantial increase in confirmed COVID-19 cases in the study will help provide a more reliable conclusion.**

**Reviewer #1: The manuscript describes mainly negative results from a prediction model based on the symptoms to a GWAS analysis. The study makes almost no suggestive conclusions but is important to publish these results nonetheless. Most of my comments would rather focus on the "what I wish were done" rather than requirements for publication since given the methodology and results and the absence of any strong conclusions, the statements made are not unreasonable.**

We would like to thank the reviewer for taking time to assess our manuscript and his appreciation of the importance of our study.

**I found the number of cases in this study too small. This is a limitation of the data, however, I think there could me more work done to increase the cohort given the widespread access to COVID-19 patient data.**

We agree that the number of cases in our study is small. However, our study should be viewed as a proof-of-concept on the use of case prediction to increase GWAS sample sizes. The current number of cases was sufficient to show the value of this approach.

**The racial diversity is quite lacking in the data to consider the effort done in GWAS analysis of high value.**

We indeed only included individuals of European ancestry in our study. This limits the generalizability of our results to other ethnicities. However, our study is a proof-of-concept study and the lack of racial diversity does not impact our main message.

**I do not understand why a conversion into binary variables was required for each of the variables in preprocessing. A trainable model is capable of dealing with ordinal variables without any conversion. Is it due to the heterogeneity between symptom reporting in different data sets?**

This was indeed due to the heterogeneity between symptom reporting in different data sets. Symptoms were not reported on an ordinal scale in all cohorts.

**The following comments are requirements for clarification of the methodology**

**- Logistic regression models are ML models, which means that there is a training phase. A complete description of the model hyperparameters and training procedures (training/test splits) is required to assess the validity of the model.**

In order to validate logistic regression models, rather than applying training/test splits in the Lifelines cohort, we used the Helix and NTR cohorts for validation.

**There are multiple mathematical/statistical notions that need to be properly typed (italic p for p-value... etc.)**

We thank the reviewer for the valuable feedback. We have corrected the mathematical/statistical reporting according to the guidelines of Plos One in the updated manuscript.

**PLOS ONE Journal formatting**

1. Please ensure that your manuscript meets PLOS ONE's style requirements, including those for file naming. The PLOS ONE style templates can be found at

https://journals.plos.org/plosone/s/file?id=wjVg/PLOSOne_formatting_sample_main_body.pdf and

[https://journals.plos.org/plosone/s/file?id=ba62/PLOSOne_formatting_sample_title_authors_affiliations.pdf](https://eur03.safelinks.protection.outlook.com/?url=https%3A%2F%2Fjournals.plos.org%2Fplosone%2Fs%2Ffile%3Fid%3Dba62%2FPLOSOne_formatting_sample_title_authors_affiliations.pdf&data=04%7C01%7Ci.v.van.blokland%40umcg.nl%7C0e1d317ff45842ceace108d8d181e1af%7C335122f9d4f44d67a2fccd6dc20dde70%7C0%7C1%7C637489703052025935%7CUnknown%7CTWFpbGZsb3d8eyJWIjoiMC4wLjAwMDAiLCJQIjoiV2luMzIiLCJBTiI6Ik1haWwiLCJXVCI6Mn0%3D%7C2000&sdata=HmwICno4UBcZqK6XAktMaTbVIojb1D9MvM1D6PwTOQ0%3D&reserved=0)

We incorporated PLOS ONE´s style requirements into our revised manuscript.

**2.We note that the grant information you provided in the ‘Funding Information’ and ‘Financial Disclosure’ sections do not match.**

**When you resubmit, please ensure that you provide the correct grant numbers for the awards you received for your study in the ‘Funding Information’ section.**

We added a funding information section to the manuscript as following;

**Funding Information**

*Generation Scotland received core support from the Chief Scientist Office of the Scottish Government Health Directorates [CZD/16/6] and the Scottish Funding Council [HR03006] and is currently supported by the Wellcome Trust [216767/Z/19/Z]. Genotyping of the GS:SFHS samples was carried out by the Genetics Core Laboratory at the Edinburgh Clinical Research Facility, University of Edinburgh, Scotland and was funded by the Medical Research Council UK and the Wellcome Trust (Wellcome Trust Strategic Award “STratifying Resilience and Depression Longitudinally” (STRADL) Reference 104036/Z/14/Z). Recruitment to this study was facilitated by SHARE - the Scottish Health Research Register and Biobank. SHARE is supported by NHS Research Scotland, the Universities of Scotland and the Chief Scientist Office of the Scottish Government. C.H. is supported by an MRC University Unit Programme Grant MC_UU_00007/10 (QTL in Health and Disease). Salaries of the authors ETC, KMSB, SR, AB, SW, FT, XW, JMR, YWL, JTL, and NLW was supported by Helix OpCo, LLC. The funding organization did not play a role in the study design, data analysis, decision to publish, or preparation of the manuscript for commercial purposes and only provided financial support in the form of authors' salaries and/or research materials. The Lifelines Biobank initiative has been made possible by funding from the Dutch Ministry of Health, Welfare and Sport; the Dutch Ministry of Economic Affairs; the University Medical Center Groningen (UMCG the Netherlands); the University of Groningen and the Northern Provinces of the Netherlands. The generation and management of GWAS genotype data for the Lifelines Cohort Study was supported by the UMCG Genetics Lifelines Initiative (UGLI). Lifelines COVID-19 data collection was supported by the Netherlands Organization for Scientific Research (NWO): NWO Spinoza Prize (SPI 92-266 to C.W.). L.F. is supported by an NWO Corona Fast-Track grant (440.20.001), an Oncode Senior Investigator grant, a grant from the European Research Council (ERC Starting Grant agreement number 637640 ImmRisk) and an NWO VIDI grant (917.14.374) NTR Covid-19 data collection and data management was supported by NWO and Netherlands Organisation for Health Research and Development (ZonMW) grants 440.20.022 and 480-15-001/674.*

3.We note that you have indicated that data from this study are available upon request. PLOS only allows data to be available upon request if there are legal or ethical restrictions on sharing data publicly. For information on unacceptable data access restrictions, please see <http://journals.plos.org/plosone/s/data-availability#loc-unacceptable-data-access-restrictions>.

In your revised cover letter, please address the following prompts:

a) If there are ethical or legal restrictions on sharing a de-identified data set, please explain them in detail (e.g., data contain potentially identifying or sensitive patient information) and who has imposed them (e.g., an ethics committee). Please also provide contact information for a data access committee, ethics committee, or other institutional body to which data requests may be sent.

b) If there are no restrictions, please upload the minimal anonymized data set necessary to replicate your study findings as either Supporting Information files or to a stable, public repository and provide us with the relevant URLs, DOIs, or accession numbers. Please see http://www.bmj.com/content/340/bmj.c181.long for guidelines on how to de-identify and prepare clinical data for publication. For a list of acceptable repositories, please see http://journals.plos.org/plosone/s/data-availability#loc-recommended-repositories.

We will update your Data Availability statement on your behalf to reflect the information you provide.

We thank you for bringing this to our attention. We have updated our manuscript with the following information:

Due to legal restrictions on sharing the de-identified data, individual level data can not be shared on a public repository. However, data is available upon request via the research office of each individual cohort. For contact and access, see information below. All GWAS summary statistics can be downloaded from the C19HG website <https://www.covid19hg.org/results/>.

Contact information individual cohorts:

GS:SFHS cohort is available to researchers in the UK and to international collaborators through application to the GS Access Committee. GS operates a managed data access process including an online application form (http://www.gsaccess.org/) and proposals are reviewed by the GS Access Committee.

The Helix data were collected under IRB Protocol #20170748. Data are available from the corresponding author on reasonable request.

The Lifelines data analysed in this study were obtained from the Lifelines biobank, under project application number ov20_0554. Requests to access this dataset should be directed to Lifelines Research Office (research@lifelines.nl).

Requests to access the NTR dataset should be directed to [ntr.datamanagement.fgb@vu.nl](mailto:ntr.datamanagement.fgb@vu.nl).

4. PLOS requires an ORCID iD for the corresponding author in Editorial Manager on papers submitted after December 6th, 2016. Please ensure that you have an ORCID iD and that it is validated in Editorial Manager. To do this, go to ‘Update my Information’ (in the upper left-hand corner of the main menu), and click on the Fetch/Validate link next to the ORCID field. This will take you to the ORCID site and allow you to create a new iD or authenticate a pre-existing iD in Editorial Manager. Please see the following video for instructions on linking an ORCID iD to your Editorial Manager account: https://www.youtube.com/watch?v=_xcclfuvtxQ

The corresponding author has updated the ORCID iD in the Editorial Manager (ORCID iD: 0000-0002-8545-8015).

5. One of the noted authors is a group or consortium (The COVID-19 Host Genetics Initiative,). In addition to naming the author group, please list the individual authors and affiliations within this group in the acknowledgments section of your manuscript. Please also indicate clearly a lead author for this group along with a contact email address.

We have asked the COVID-19 Host Genetics Initiative consortium about this request who informed us that a banner author is sufficient to represent the involvement of the consortium in our study. We do list author contributions in the consortium section of the manuscript. For full details, such as contact information and affiliations of its hundreds of members, we refer to the C19HG website: <https://www.covid19hg.org/acknowledgements/>.

6. We note that you have included the phrase “data not shown” in your manuscript. Unfortunately, this does not meet our data sharing requirements. PLOS does not permit references to inaccessible data. We require that authors provide all relevant data within the paper, Supporting Information files, or in an acceptable, public repository. Please add a citation to support this phrase or upload the data that corresponds with these findings to a stable repository (such as Figshare or Dryad) and provide and URLs, DOIs, or accession numbers that may be used to access these data. Or, if the data are not a core part of the research being presented in your study, we ask that you remove the phrase that refers to these data.

We thank you for bringing this to our attention. In the caption of **Figure S4**, we have inadvertently included the phrase (not shown). We have rewritten the sentence at hand, in order to meet the data sharing requirements of PLOS ONE from the following:

*The same diseases are Bonferroni significant and not significant, respectively, when applying generalised linear models with “age”, “sex” and “bmi” as confounding variables (not shown).*

To the updated line:

*Applying generalised linear models with “age”, “sex” and “bmi” as covariates, instead of Fishers exact tests, does not change which diseases are Bonferroni significant and which are not.*

7.Thank you for stating the following in the Competing Interests section:

"ETC, KMSB, SR, AB, SW, FT, XW, JMR, YWL, JTL, and NLW are employees of Helix. All other authors declare no financial or non-financial conflict of interest."

We note that one or more of the authors are employed by a commercial company: Helix

a) Please provide an amended Funding Statement declaring this commercial affiliation, as well as a statement regarding the Role of Funders in your study. If the funding organization did not play a role in the study design, data collection and analysis, decision to publish, or preparation of the manuscript and only provided financial support in the form of authors' salaries and/or research materials, please review your statements relating to the author contributions, and ensure you have specifically and accurately indicated the role(s) that these authors had in your study. You can update author roles in the Author Contributions section of the online submission form.

Please also include the following statement within your amended Funding Statement.

“The funder provided support in the form of salaries for authors [insert relevant initials], but did not have any additional role in the study design, data collection and analysis, decision to publish, or preparation of the manuscript. The specific roles of these authors are articulated in the ‘author contributions’ section.”

If your commercial affiliation did play a role in your study, please state and explain this role within your updated Funding Statement.

b) Please also provide an updated Competing Interests Statement declaring this commercial affiliation along with any other relevant declarations relating to employment, consultancy, patents, products in development, or marketed products, etc.

Within your Competing Interests Statement, please confirm that this commercial affiliation does not alter your adherence to all PLOS ONE policies on sharing data and materials by including the following statement: "This does not alter our adherence to PLOS ONE policies on sharing data and materials.” (as detailed online in our guide for authors http://journals.plos.org/plosone/s/competing-interests) . If this adherence statement is not accurate and there are restrictions on sharing of data and/or materials, please state these. Please note that we cannot proceed with consideration of your article until this information has been declared.

We have updated the funding and competing interests statement with the changes highlighted below and added the updated statements to the revised manuscript.

**Updated funding statement**:

*Generation Scotland received core support from the Chief Scientist Office of the Scottish Government Health Directorates [CZD/16/6] and the Scottish Funding Council [HR03006] and is currently supported by the Wellcome Trust [216767/Z/19/Z]. Genotyping of the GS:SFHS samples was carried out by the Genetics Core Laboratory at the Edinburgh Clinical Research Facility, University of Edinburgh, Scotland and was funded by the Medical Research Council UK and the Wellcome Trust (Wellcome Trust Strategic Award “STratifying Resilience and Depression Longitudinally” (STRADL) Reference 104036/Z/14/Z). Recruitment to this study was facilitated by SHARE - the Scottish Health Research Register and Biobank. SHARE is supported by NHS Research Scotland, the Universities of Scotland and the Chief Scientist Office of the Scottish Government. C.H. is supported by an MRC University Unit Programme Grant MC_UU_00007/10 (QTL in Health and Disease). Salaries of the authors ETC, KMSB, SR, AB, SW, FT, XW, JMR, YWL, JTL, and NLW was supported by Helix OpCo, LLC. The funding organization did not play a role in the study design, data analysis, decision to publish, or preparation of the manuscript for commercial purposes and only provided financial support in the form of authors' salaries and/or research materials. The Lifelines Biobank initiative has been made possible by funding from the Dutch Ministry of Health, Welfare and Sport; the Dutch Ministry of Economic Affairs; the University Medical Center Groningen (UMCG the Netherlands); the University of Groningen and the Northern Provinces of the Netherlands. The generation and management of GWAS genotype data for the Lifelines Cohort Study was supported by the UMCG Genetics Lifelines Initiative (UGLI). Lifelines COVID-19 data collection was supported by the Netherlands Organization for Scientific Research (NWO): NWO Spinoza Prize (SPI 92-266 to C.W.). L.F. is supported by an NWO Corona Fast-Track grant (440.20.001), an Oncode Senior Investigator grant, a grant from the European Research Council (ERC Starting Grant agreement number 637640 ImmRisk) and an NWO VIDI grant (917.14.374) NTR Covid-19 data collection and data management was supported by NWO and Netherlands Organisation for Health Research and Development (ZonMW) grants 440.20.022 and 480-15-001/674.*

**Updated competing interests statement**: *The authors ETC, KMSB, SR, AB, SW, FT, XW, JMR, YWL, JTL, and NLW are employees of Helix OpCo, LLC, which is a provider of COVID-19 testing services. This does not alter the authors' and institutions' adherence to PLOS ONE policies on sharing data and materials. All other authors declare no financial or non-financial conflict of interest.*

Please include both an updated **Funding Statement** and **Competing Interests Statement** in your cover letter. We will change the online submission form on your behalf.

Please know it is PLOS ONE policy for corresponding authors to declare, on behalf of all authors, all potential competing interests for the purposes of transparency. PLOS defines a competing interest as anything that interferes with, or could reasonably be perceived as interfering with, the full and objective presentation, peer review, editorial decision-making, or publication of research or non-research articles submitted to one of the journals. Competing interests can be financial or non-financial, professional, or personal. Competing interests can arise in relationship to an organization or another person. Please follow this link to our website for more details on competing interests: http://journals.plos.org/plosone/s/competing-interests
