# Supplementary material for: Using symptom-based case predictions to identify host genetic factors that contribute to COVID-19 susceptibility
Source: PLoS One. 2021 Aug 11;16(8):e0255402. doi: 10.1371/journal.pone.0255402 (PMC8357137; doi:10.1371/journal.pone.0255402)
Supplement: Attachment — Submitted filename: Response_to_reviewers_PONE-D-20-38159-vanBlokland_20210628.docx [file pone.0255402.s014.docx]

**PONE-D-20-38159**

**Authors:** van Blokland *et al*.

**Response to reviewers**

We thank the editors and and reviewers for taking the time to consider our manuscript. Please see below point-by-point answer to the comments raised by the academic editor and reviewer.

**Journal Requirements:**

**Please review your reference list to ensure that it is complete and correct. If you have cited papers that have been retracted, please include the rationale for doing so in the manuscript text, or remove these references and replace them with relevant current references. Any changes to the reference list should be mentioned in the rebuttal letter that accompanies your revised manuscript. If you need to cite a retracted article, indicate the article’s retracted status in the References list and also include a citation and full reference for the retraction notice.**

**We have updated reference 11 from the following**

Mc Intyre K, Lanting P, Deelen P, Wiersma H, Vonk JM, Ori AP, et al. The Lifelines COVID-19 Cohort: a questionnaire-based study to investigate COVID-19 infection and its health and societal impacts in a Dutch population-based cohort. medRxiv. 2020 Jun 24;2020.06.19.20135426.

**To the updated reference:**

Mc Intyre K, Lanting P, Deelen P, Wiersma HH, Vonk JM, et al. Lifelines COVID-19 cohort: investigating COVID-19 infection and its health and societal impacts in a Dutch population-based cohort. BMJ Open. 2021 Mar 17;11(3):e044474. doi: 10.1136/bmjopen-2020-044474.

**Reviewer #2: This manuscript reports symptom-based predictive phenotypes as proxies for COVID-19 to understand genetic susceptibility to COVID-19. The strategy is well thought out and there are no problems. However, it is not clear whether or not differences due to the genetic background of the population have been taken into account. Are the eight genome-wide significantly different variants for viral infections selected from the NHGRI-EBI GWAS Catalog from a valid population for this comparison? Are GWAS meta-analyses of cohorts containing different genetic backgrounds valid? Should the current findings be considered valid only for specific populations? For example, rs5798227 associated with COVID-19 seems to be present in most Asians. It would be better if there was mention of the differences in genetic background among populations and how this affects the prediction model.**

Thank you for your remark, this is a valid consideration with two potential implications. The first is that the GWASs on the other viral infections might have been performed on non-European populations. If that were to be the case, a direct comparison of lead or top variants might not be straightforward. To ensure that this is not the case for the overlapping variants between our predicted COVID19 GWAS and the other viral infections we double checked this. All these GWASes are primarily conducted on Europeans. The cohorts that we used are also primarily of European descent so these lead variants can be compared, and a meta-analysis could be conducted without special considerations. We have included this statement in the manuscript in the description of **Supplementary Figure 2.**

Secondly, there could indeed be a difference in minor allele frequencies among different populations. This could indicate that some populations might be slightly more or less susceptible to an infection. However the association is still relevant to other populations, this is extensively discussed here: <https://www.nature.com/articles/s41586-020-2818-3>
